# Transcript abundance of stromal and thecal cell related genes during bovine ovarian development

**DOI:** 10.1371/journal.pone.0213575

**Published:** 2019-03-11

**Authors:** Nicholas Hatzirodos, Katja Hummitzsch, Helen F. Irving-Rodgers, James Breen, Viv E. A. Perry, Richard A. Anderson, Raymond J. Rodgers

**Affiliations:** 1 Discipline of Obstetrics and Gynaecology, School of Medicine, Robinson Research Institute, University of Adelaide, Adelaide, South Australia, Australia; 2 School of Medical Science, Griffith University, Gold Coast Campus, Gold Coast, Queensland, Australia; 3 South Australian Health and Medical Research Institute (SAHMRI), Adelaide, South Australia, Australia; 4 University of Adelaide Bioinformatics Hub, Adelaide, South Australia, Australia; 5 School of Veterinary Medicine and Science, University of Nottingham, Sutton Bonington, Leicestershire, United Kingdom; 6 Medical Research Council Centre for Reproductive Health, University of Edinburgh, Queen’s Medical Research Institute, Edinburgh, United Kingdom; Colorado State University, UNITED STATES

## Abstract

Movement and expansion of mesonephric-derived stroma appears to be very important in the development of the ovary. Here, we examined the expression of 24 genes associated with stroma in fetal ovaries during gestation (n = 17; days 58–274) from *Bos taurus* cattle. RNA was isolated from ovaries for quantitative RT-PCR. Expression of the majority of genes in TGFβ signalling, stromal transcription factors (*NR2F2*, *AR)*, and some stromal matrix genes (*COL1A1*, *COL3A1* and *FBN1*, but not *FBN3*) showed a positive linear increase with gestational age. Expression of genes associated with follicles (*INSL3*, *CYP17A1*, *CYP11A1* and *HSD3B1*), was low until mid-gestation and then increased with gestational age. *LHCGR* showed an unusual bimodal pattern; high levels in the first and last trimesters. *RARRES1* and *IGFBP3* also increased with gestational age. To relate changes in gene expression in stromal cells with that in non stromal cells during development of the ovary we combined the data on the stromal genes with another 20 genes from non stromal cells published previously and then performed hierarchical clustering analysis. Three major clusters were identified. Cluster 1 genes (*GATA4*, *FBN3*, *LHCGR*, *CYP19A1*, *ESR2*, *OCT4*, *DSG2*, *TGFB1*, *CCND2*, *LGR5*, *NR5A1*) were characterised by high expression only in the first trimester. Cluster 2 genes (*FSHR*, *INSL3*, *HSD3B1*, *CYP11A1*, *CYP17A1*, *AMH*, *IGFBP3*, *INHBA*) were highly expressed in the third trimester and largely associated with follicle function. Cluster 3 (*COL1A1*, *COL3A1*, *FBN1*, *TGFB2 TGFB3*, *TGFBR2*, *TGFBR3*, *LTBP2*, *LTBP3*, *LTBP4*, *TGFB1I1*, *ALDH1A1*, *AR*, *ESR1*, *NR2F2*) had much low expression in the first trimester rising in the second trimester and remaining at that level during the third trimester. Cluster 3 contained members of two pathways, androgen and TGFβ signalling, including a common member of both pathways namely the androgen receptor cofactor TGFβ1 induced transcript 1 protein (*TGFB1I1*; hic5). *GATA4*, *FBN3* and *LHCGR*, were highly correlated with each other and were expressed highly in the first trimester during stromal expansion before follicle formation, suggesting that this could be a critical phase in the development of the ovarian stroma.

## Introduction

The mammalian ovary undergoes many different cell processes during its development [1]. The somatic granulosa cells of follicles and the surface epithelial cells, except at the base of the ovary, are derived from a common precursor, the gonadal ridge-epithelial-like (GREL) cell. This precursor cell very likely originates from the surface epithelial cells of the mesonephros and replicates to form the gonadal ridge. The oogonia and oocytes arise from primordial germ cells (PGC) that migrate from as far away as the yolk sac into the mesonephros from where they are incorporated into the gonadal ridge [2]. During their migration, the PGCs start to proliferate—a process which is continued after colonising the gonad. In the developing ovary, the germ cells firstly replicate (oogonia), then subsequently enter meiosis and arrest in the dictyate stage of prophase I (oocytes) until ovulation.

The ovarian stroma arises from the mesonephric stroma, as does the ovarian vasculature. This stroma penetrates the developing ovary and branches between the mass of GREL cells and PGCs/oogonia to form ovigerous cords [1]. Subsequently the ovigerous cords break into smaller and smaller groups of GREL cells and PGCs/oogonia until the first primordial follicles are formed. The processes involved in this breakdown are not well understood but certainly stroma penetrates in between these groups of cells during this process. The stroma spreads laterally below the surface thus partitioning some GRELs onto the surface of the ovary which then develop into a specialised epithelial layer. The expansion of cortical stroma is greatest in early gestation due to stromal cell proliferation [3].

The stroma is rich in stromal matrices such as decorin, versican, collagens type 1 and III, latent TGFβ binding proteins (LTBP) and fibrillins [1,4–6]. However, the matrix is not static during ovarian development. Fibrillin 3 is expressed in the first trimester only, and then in trimester 2 fibrillins 1 and 2 are expressed [6]. This maturational change in fibrillin types also occurs when fetal fibroblasts are cultured in vitro [7] and since fibrillins bind LTBPs and regulate bioavailability of TGFβ in tissues, presumably the action of TGFβ changes during fetal development of the ovary.

The importance of stroma in the ovary is best illustrated in polycystic ovary syndrome (PCOS) [8,9]. In the PCOS ovary all stromal compartments are different to normal ovaries. The polycystic ovaries have fifty per cent more ovarian capsule or tunica albuginea containing more collagen [9]. Their thickness of the cortical stromal is increased by one third, and the subcortical stroma, whether deep cortical or medullary, by five fold [9]. Functionally, the specialised stromal theca interna layer that develops around antral follicles also behaves differently in PCOS, with elevated capacity to produce steroid hormones [10,11]. The consequences of expanded stroma are well known in fibrosis, where TGFβ stimulates stromal fibroblast replication and deposition of collagen [12,13]. It has therefore logically been proposed that altered TGFβ activity in stroma is involved in development of polycystic ovaries [6]. However, this suggestion [6] was from a study of human and bovine fetal ovaries, not adult ovaries where polycystic ovaries can be observed [6]. This is reasonable as there is evidence for a fetal origin of PCOS that comes from a variety of sources now [14–18] and it is widely agreed now that events in fetal life affect the predisposition to PCOS in later life. The formation of the fetal ovarian stroma may be a critical event in this process.

Little is known about ovarian stroma, much less its development in the fetal ovary. In a morphometric study of the developing bovine ovary [3] it was found that during growth of cortex and medulla across gestation, the rate of cortical expansion slowed and the proliferation index and numerical density of proliferating cells in stroma significantly decreased. However, the proportion of stroma in the cortex significantly increased as the non stromal cells ceased dividing. Thus the stroma appears to be active throughout development. To further understand the role of stromal cells and matrices in the process of ovarian development and working on the hypothesis that development of the ovary is under genetic control we examined the expression patterns of genes related to TGFβ signalling, extracellular matrix and androgen synthesis throughout gestation in fetal ovaries. Genes related to germ cells, GREL cells and granulosa cells have been discussed previously [19] but analyses of the combined data sets have additionally been undertaken and presented here. We used the bovine ovary, which is similar to the human ovary [20,21], including fetal ovary development [6] and a summary of gene expression in the ovary was also informative in our selection of genes [22].

## Materials and methods

### Tissues

Fetuses of pregnant *Bos taurus* cows were collected at T&R Pastoral abattoir at Murray Bridge, SA, Australia and then transported on ice to the laboratory (n = 17). Crown-rump length (CRL) was measured to estimate gestational age [23]. All ovaries were frozen at -80°C for subsequent RNA analyses.

### Gender determination

To confirm the gender of young fetuses (CRL < 8 cm), genomic DNA was extracted from the tail samples using the Wizard SV Genomic DNA Purification System (Promega Australia, Alexandria, NSW, Australia) according to the manufacturer’s instructions. Genomic DNA was amplified with a primer pair (sense primer: 5’-TCACTCCTGCAAAAGGAGCA-3’, antisense primer: 5’-TTATTGTGGCCCAGGCTTG-3’), specific for a region in the SRY-determining sequence, and primers specific for the 18S ribosomal RNA gene sequence [24] in separate reactions. SRY product sequences were verified by automated sequencing (3730 DNA analyser; Applied Biosystems, Mulgrave, VIC, Australia).

### RNA extraction, cDNA synthesis and quantitative real time PCR

All fetal gonad samples for RNA sample extraction were homogenised with 1 ml of Trizol (Cat # 15596–026, Thermo Fisher Scientific, Waltham, MA, USA) each for two 10 s cycles at 3,500 rpm in a PowerLyzer 24 Bench Top Bead-Based Homogenizer (MoBio, Carlsbad, Ca, USA). The samples were then processed according to the standard Trizol protocol and resuspended in 30 μl of nuclease free H_2_O. Ten μg or less of each sample was treated with 2 U of DNAse 1 for 20 min at 37° C and the enzyme removed using DNAse inactivation reagent (Thermo Fisher Scientific). Two hundred ng of DNAse-treated RNA was used for reverse transcription reactions with or without Superscript RT III (Thermo Fisher Scientific) to generate cDNA or negative control to detect genomic contaminant respectively.

Primer assays were designed where possible to span intron-exon junctions or from one exon to another for quantitative real time PCR (qRT-PCR) using the free web-based software programs, Primer3 plus (Rozen and Skaletsky) and Net primer (PREMIER Biosoft, Palo Alto, CA, USA), based on the Reference RNA sequences available in NCBI (S1 Table). The cDNA was diluted from 1 in 4 to 1in 1000 and pooled from 10 samples to generate 5 standards which were used to establish a standard curve for testing primer combinations for quantitative real time PCR. Only those assays which gave a single sharp peak by melt curve analysis and achieved an amplification efficiency of 0.9–1.1 and an R^2^ value ≥ 0.98 were used for quantitation of transcript abundance.

The reactions were performed in duplicate on a Fluidigm Biomark HD instrument (San Francisco, CA, USA) using the following protocol. The reaction started with a pre-amplification step consisting of a 95°C hold for 10 min, followed by 12 cycles of 95°C for 15 s and 60°C for 4 min each using 50 nM of each primer and 2.5 μl of cDNA in 10 μl. The amplified product was then diluted 1 in 5 and added in 0.05 μl to the final reaction volume of 0.1 μl in a 48x48 plate which contained 500 nM of each primer per assay. The final amplification conditions were a 60 s activation step at 95°C, followed by 30 cycles of 96°C denaturation for 5 s and 60°C annealing/extension for 20 s using SsoFast EvaGreen Supermix with Low ROX (Biorad, Hercules, CA, USA) which contains a fluorescent intercalating agent for measuring amplification. The expression values for each gene were determined as the mean of the ratio of 2^-Δ Ct^ for the target gene to the mean of *RPL32* and *PPIA* because this gene combination was determined to be the most stable across all samples out of *RPL32*, *PPIA*, *ACTB* and *GAPDH* with a value of 0.056 using the Normfinder program.

### Statistical analyses

ANOVA and post hoc statistical calculations using Tukey’s test were performed using GraphPad Prism version 6.00 (GraphPad Software Inc., La Jolla, CA, USA) following log transformation where appropriate to normalise the raw data distribution. Pearson’s correlations across all target genes and samples were performed on the data in Partek Genomics Suite (St Louis, MI, USA). Hierarchical clustering by gene only was also performed on the data using the Euclidian algorithm for dissimilarity with average linkage in Partek to generate a heatmap of relative transcript abundance. Prior to clustering, the raw data were first normalised by adjusting the mean expression across all samples for each gene to zero and the standard deviation to one. After correlation values between genes were identified, a network graph was plotted using the qgraph R package [25] and illustrated as an adjacent matrix plot.

## Results

Due to the large number of genes analysed, we will only describe the expression profiles of genes associated with stromal and thecal cells in the following paragraphs.

### TGFβ signalling pathway

**Fig 1:**
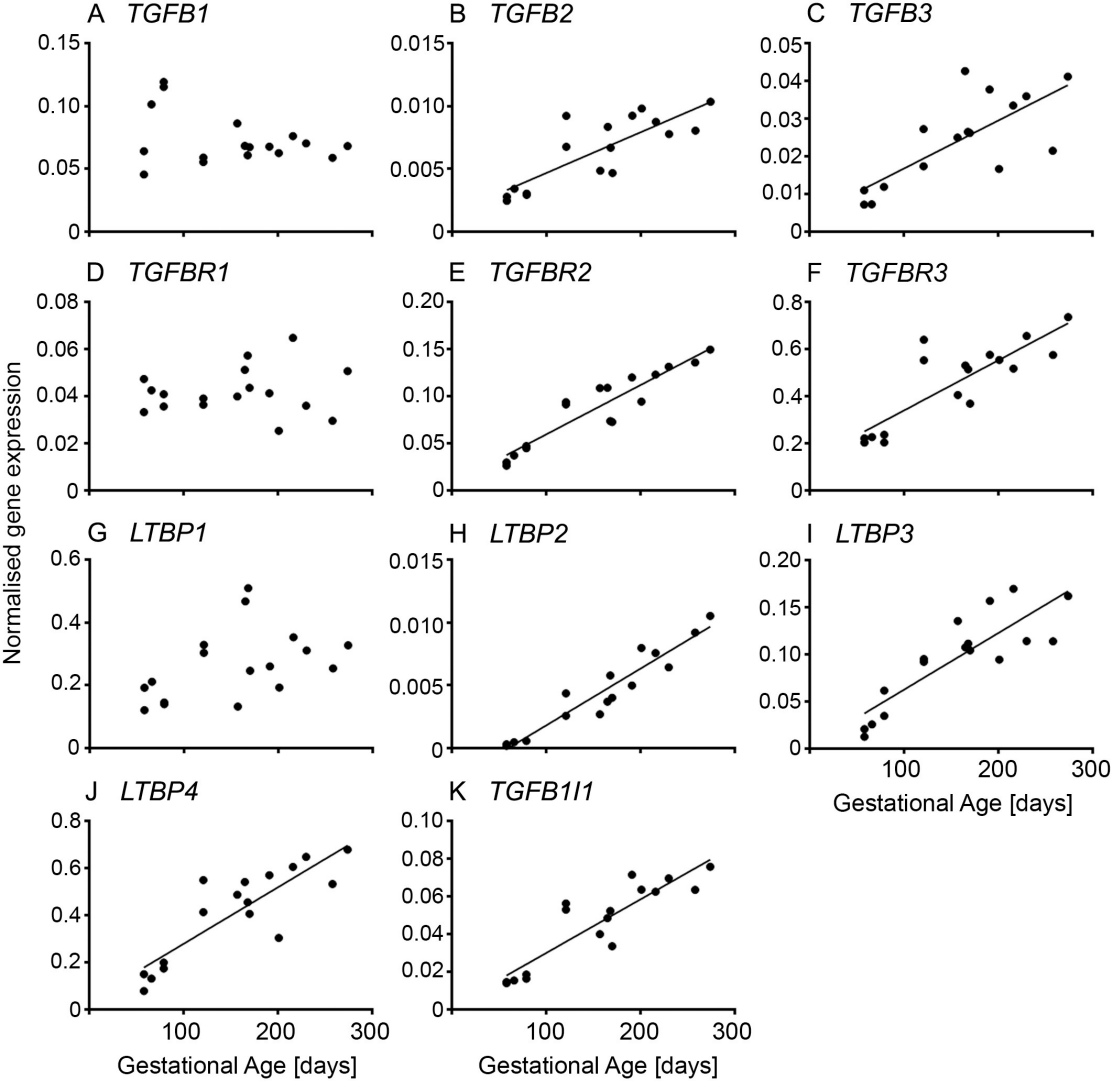
TGF-β signalling pathway in fetal ovarian development. Expression levels of genes for each animal are plotted against gestational age in days (n = 16 or 17 animals).

The majority of genes involved in TGFβ signalling (Fig 1 and S1 Fig) increased throughout fetal ovarian development and showed a positive linear responses with gestational age, apart from *TGFB1* (Fig 1A and S1A Fig), *TGFBR1* (Fig 1D and S1D Fig) and *LTBP1* (Fig 1G and S1G Fig). The expression of these three genes was unchanged throughout gestation.

### Stromal matrix and stromal markers

**Fig 2:**
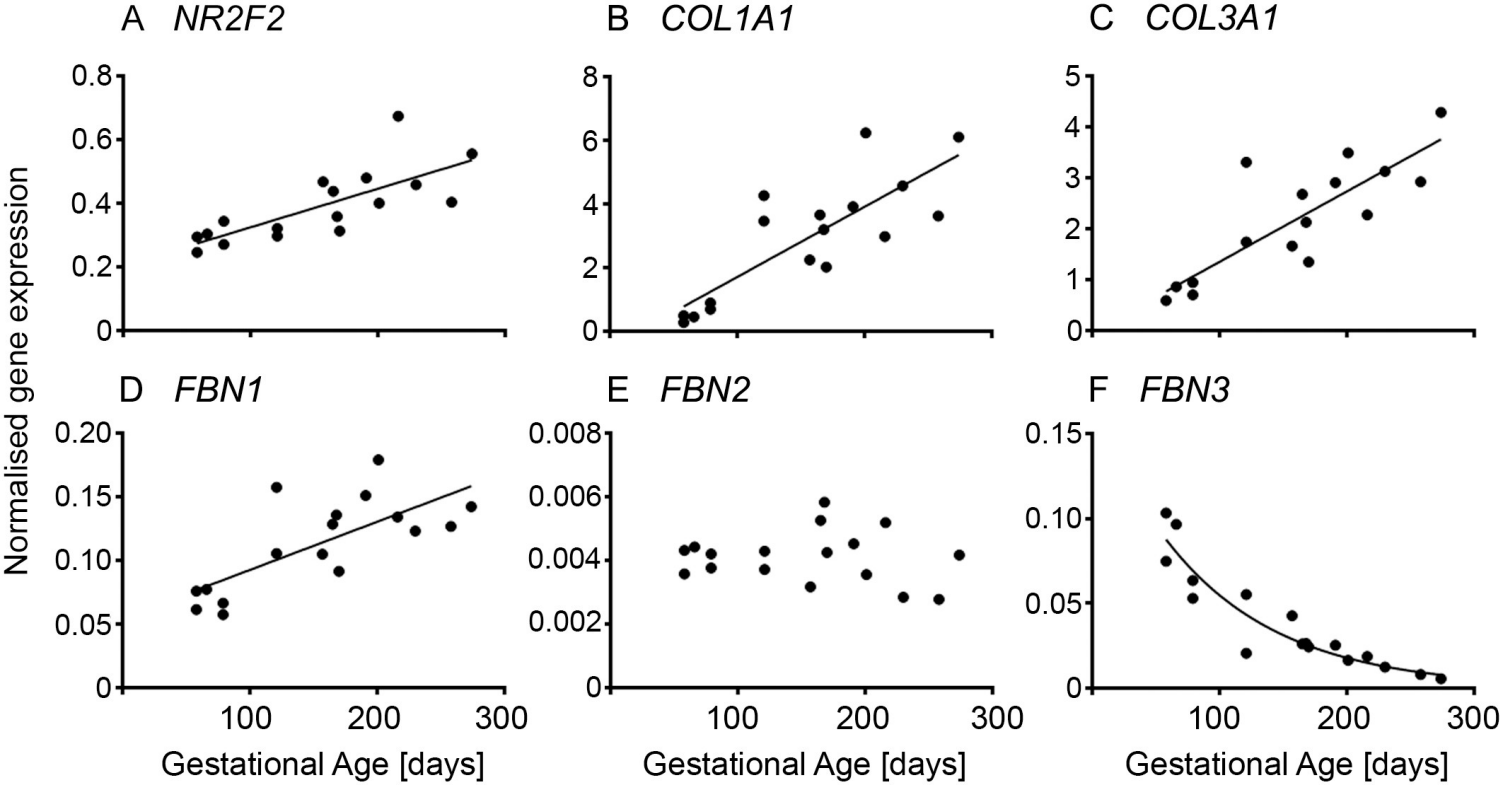
Stromal matrix and marker in fetal ovarian development. Expression levels of genes for each animal are plotted against gestational age in days (n = 16 or 17 animals).

The expression of *NR2F2* (*COUP-TFII*; Fig 2A and S2A Fig), a specific stromal cell marker, increased linearly with gestational age. This coincided with the observed increase to high levels in the expression of extracellular matrix genes *COL1A1*, *COL3A1* and *FBN1* (Fig 2B–2D and S2B–S2D Fig) during gestation. In contrast to *FBN1*, *FBN2* (Fig 2E and S2E Fig) expression remained unchanged and *FBN3* (Fig 2F and S2F Fig) declined drastically with age as shown previously [6].

### Steroidogenesis and growth factor signalling

**Fig 3:**
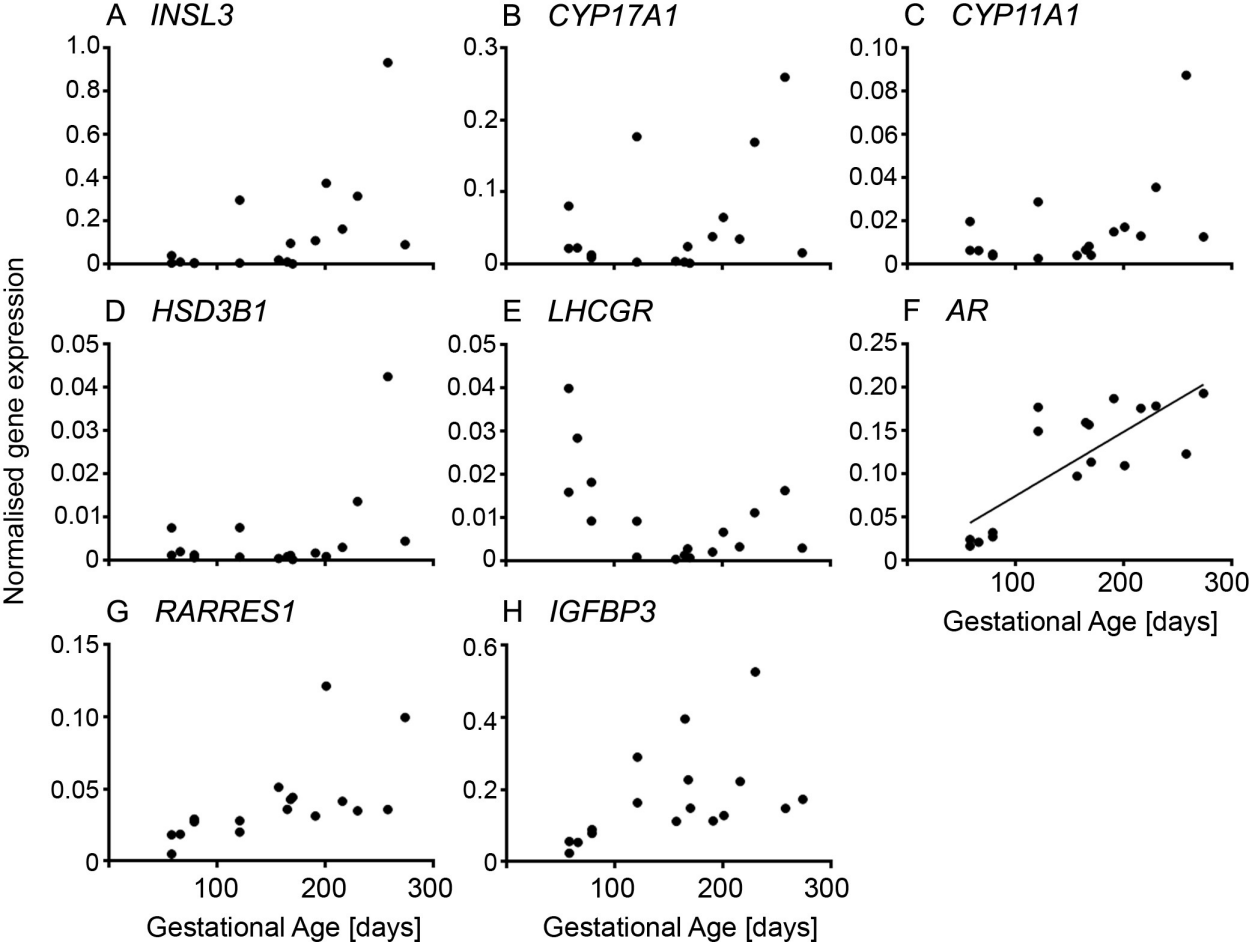
Steroidogenesis and growth factor signalling in fetal ovarian development. Expression levels of genes for each animal are plotted against gestational age in days (n = 16 or 17 animals).

The expression of *INSL3* (Fig 3A and S3A Fig), a peptide hormone involved in regulating steroidogenesis [26], and enzymes of the androgen synthesis pathway, *CYP17A1*, *CYP11A1* and *HSD3B1* (Fig 3B–3D and S3B–S3D Fig), were low until mid-gestation and then increased with gestational age. *LHCGR* (Fig 3E and S3E Fig) was highly expressed early in gestation, declined rapidly to a low point in mid-gestation and then slightly increased again towards the end of gestation. The expression of *AR*, *RARRES1* and *IGFBP3* (Fig 3F–3H and S3F–S3H Fig) increased with gestational age.

### Relationships between functional groups of genes

Pearson’s correlation analysis were conducted and revealed correlations between genes within and between functional groups. A few examples of scatter plots of the mRNA levels between different genes are shown in S4 Fig. Most of the examined members of the TGFβ signalling showed strong positive correlation between each other, except for *TGFB1* and *TGFBR1* (Table 1). *TGFB1* had only a strong negative correlation with *TGFBR3*, and *TGFBR1* a strong positive correlation to *LTBP1*. Furthermore, all genes of the TGFβ signalling, except for *TGFB1* and *TGFBR1*, were strongly positively correlated with the stromal cell marker *NR2F2* and the extracellular matrix genes *COL1A1*, *COL3A1* and *FBN1*, and strongly negatively with *FBN3*. *TGFBR1* and *LTBP1* were strongly correlated with *FBN2*. *TGFB1* was only negatively correlated with *FBN1*. TGFβ signalling seemed to be not correlated with the enzymes of androgen synthesis, except for *LTBP2* which was positively correlated with *CYP11A1*. Only *TGFBR2*, *LTBP2* and *TGFB1I1* had a positive correlation with *INSL3*. *TGFB2*, *TGFB3*, *TGFBR2*, *TGFBR3*, *LTBP3*, *LTBP4* and *TGFB1I1* were strongly negatively correlated with *LHCGR*. All TGFβ signalling genes examined, were positively correlated with *AR*, except for *TGFB1* and *TGFBR1*, and were positively correlated with *IGFBP3*, except for *TGFB1*, *TGFBR1*, *LTPB2* and *LTBP3*. *TGFB2*, *TGFBR2*, *TGFBR3*, *LTBP2* and *TGFB1I1* are also positively correlated with *RARRES1*.

**Table 1:** Pearson’s correlation coefficients between expression of genes in TGF-β signalling with other genes in TGF-β signalling, stromal matrix and marker and thecal cells and gestational age. The darker the background colour the more significant the correlation is. Blue indicates a negative correlation and red indicates a positive correlation. (*P*-values: a < 0.05, b < 0.01, c < 0.001, d < 0.0001).

		TGF-β Signalling										
		*TGFB1*	*TGFB2*	*TGFB3*	*TGFBR1*	*TGFBR2*	*TGFBR3*	*LTBP1*	*LTBP2*	*LTBP3*	*LTBP4*	*TGFB1I1*
Gestational age		-0.330	**0.818**^**d**^	**0.758**^**c**^	0.121	**0.838**^**d**^	**0.930**^**d**^	0.454	**0.957**^**d**^	**0.857**^**d**^	**0.855**^**d**^	**0.895**^**d**^
TGF-β Signalling	***TGFB1***		-0.441	-0.282	-0.033	-0.298	**-0.499**^**a**^	-0.418	-0.395	-0.228	-0.334	-0.452
	***TGFB2***	-0.441		**0.720**^**b**^	0.092	**0.861**^**c**^	**0.945**^**c**^	**0.575**^**a**^	**0.863**^**c**^	**0.796**^**c**^	**0.796**^**c**^	**0.954**^**c**^
	***TGFB3***	-0.282	**0.720**^**b**^		0.418	**0.832**^**c**^	**0.764**^**c**^	**0.665**^**b**^	**0.630**^**b**^	**0.850**^**c**^	**0.920**^**c**^	**0.776**^**c**^
	***TGFBR1***	-0.033	0.092	0.418		0.110	0.076	**0.604**^**a**^	0.107	0.34	0.279	0.082
	***TGFBR2***	-0.298	**0.861**^**c**^	**0.832**^**c**^	0.110		**0.900**^**c**^	0.457	**0.857**^**c**^	**0.902**^**c**^	**0.943**^**c**^	**0.928**^**c**^
	***TGFBR3***	**-0.499**^**a**^	**0.945**^**c**^	**0.764**^**c**^	0.076	**0.900**^**c**^		**0.617**^**b**^	**0.849**^**c**^	**0.801**^**c**^	**0.883**^**c**^	**0.968**^**c**^
	***LTBP1***	-0.418	**0.575**^**a**^	**0.665**^**b**^	**0.604**^**a**^	0.457	**0.617**^**b**^		0.469	**0.497**^**a**^	**0.617**^**b**^	**0.554**^**a**^
	***LTBP2***	-0.395	**0.863**^**c**^	**0.630**^**b**^	0.107	**0.857**^**c**^	**0.849**^**c**^	0.469		**0.784**^**c**^	**0.758**^**c**^	**0.893**^**c**^
	***LTBP3***	-0.228	**0.796**^**c**^	**0.850**^**c**^	0.340	**0.902**^**c**^	**0.801**^**c**^	**0.497**^**a**^	**0.784**^**c**^		**0.916**^**c**^	**0.864**^**c**^
	***LTBP4***	-0.334	**0.796**^**c**^	**0.920**^**c**^	0.279	**0.943**^**c**^	**0.883**^**c**^	**0.617**^**b**^	**0.758**^**c**^	**0.916**^**c**^		**0.891**^**c**^
	***TGFB1I1***	-0.452	**0.954**^**c**^	**0.776**^**c**^	0.082	**0.928**^**c**^	**0.968**^**c**^	**0.554**^**a**^	**0.893**^**c**^	**0.864**^**c**^	**0.891**^**c**^	
Stromal Matrix and Marker	***NR2F2***	-0.049	**0.652**^**b**^	**0.740**^**c**^	0.443	**0.786**^**c**^	**0.590**^**a**^	0.352	**0.691**^**b**^	**0.855**^**c**^	**0.752**^**c**^	**0.695**^**b**^
	***COL1A1***	-0.436	**0.944**^**c**^	**0.661**^**b**^	-0.077	**0.822**^**c**^	**0.936**^**c**^	**0.479**	**0.859**^**c**^	**0.718**^**b**^	**0.745**^**c**^	**0.926**^**c**^
	***COL3A1***	-0.398	**0.959**^**c**^	**0.676**^**b**^	-0.017	**0.863**^**c**^	**0.946**^**c**^	**0.488**^**a**^	**0.884**^**c**^	**0.737**^**c**^	**0.761**^**c**^	**0.930**^**c**^
	***FBN1***	**-0.503**^**a**^	**0.945**^**c**^	**0.572**^**a**^	0.044	**0.735**^**c**^	**0.870**^**c**^	**0.533**^**a**^	**0.799**^**c**^	**0.712**^**b**^	**0.655**^**b**^	**0.887**^**c**^
	***FBN2***	-0.039	0.057	0.249	**0.815**^**c**^	-0.147	-0.043	**0.632**^**b**^	-0.071	0.113	0.038	-0.036
	***FBN3***	0.251	**-0.837**^**c**^	**-0.726**^**c**^	-0.034	**-0.856**^**c**^	**-0.844**^**c**^	**-0.485**^**a**^	**-0.868**^**c**^	**-0.842**^**c**^	**-0.821**^**c**^	**-0.866**^**c**^
Steroidogenesis and growth factor signalling	***INSL3***	-0.319	0.462	0.041	-0.413	**0.489**^**a**^	0.459	0.063	**0.624**^**b**^	0.250	0.294	**0.498**^**a**^
	***CYP17A1***	-0.357	0.323	-0.063	-0.411	0.338	0.390	0.034	0.407	0.069	0.187	0.365
	***CYP11A1***	-0.338	0.333	0.026	-0.356	0.428	0.379	0.047	**0.508**^**a**^	0.160	0.257	0.395
	***HSD3B1***	-0.269	0.210	-0.006	-0.321	0.375	0.284	0.018	0.433	0.102	0.223	0.293
	***LHCGR***	0.060	**-0.570**^**a**^	**-0.717**^**b**^	-0.159	**-0.607**^**b**^	**-0.579**^**a**^	-0.425	-0.459	**-0.769**^**c**^	**-0.719**^**b**^	**-0.597**^**a**^
	***AR***	-0.455	**0.898**^**c**^	**0.868**^**c**^	0.298	**0.866**^**c**^	**0.942**^**c**^	**0.720**^**b**^	**0.762**^**c**^	**0.874**^**c**^	**0.930**^**c**^	**0.929**^**c**^
	***RARRES1***	-0.094	**0.579**^**a**^	0.336	-0.108	**0.489**^**a**^	**0.490**^**a**^	0.082	**0.680**^**b**^	0.480	0.348	**0.537**^**a**^
	***IGFBP3***	-0.224	**0.541**^**a**^	**0.646**^**b**^	0.156	**0.567**^**a**^	**0.646**^**b**^	**0.646**^**b**^	0.416	0.441	**0.648**^**b**^	**0.568**^**a**^

Looking at the relationships between extracellular matrix genes, we found a very strong correlation between the three genes *COL1A1*, *COL3A1* and *FBN1* and a strong positive correlation of these three genes with *NR2F2* (Table 2). All four genes were strongly negatively correlated with *FBN3*. *FBN2* showed no correlation with any of the other extracellular matrix genes, but a negative correlation with the steroidogenic genes *INSL3*, *CYP11A1*, *CYP17A1* and *HSD3B1*. *NR2F2*, *COL1A1*, *COL3A1* and *FBN1* were negatively correlated with *LHCGR* but positively with *AR*, whereas *FBN3* showed the reverse pattern for these two genes. *COL1A1*, *COL3A1* and *FBN1* were positively correlated with *RARRES1*. Additionally the collagen genes also showed a positive correlation with *IGFBP3*. *FBN3* on the other hand was negatively correlated with *RARRES1* and *IGFBP3*.

**Table 2:** Pearson’s correlation coefficients between levels of expression of genes for stromal matrix and marker with other genes for stromal matrix and marker and thecal cells and gestational age. The darker the background colour the more significant the correlation is. Blue indicates a negative correlation and red indicates a positive correlation. (*P*-values: a < 0.05, b < 0.01, c < 0.001, d < 0.0001).

		Stromal Matrix and Marker					
		*NR2F2*	*COL1A1*	*COL3A1*	*FBN1*	*FBN2*	*FBN3*
Gestational age		**0.754**^**c**^	**0.816**^**d**^	**0.843**^**d**^	**0.727**^**c**^	-0.114	**-0.890**^**d**^
Stromal Matrix and Marker	***NR2F2***		**0.543**^**a**^	**0.585**^**a**^	**0.527**^**a**^	0.122	**-0.631**^**b**^
	***COL1A1***	**0.543**^**a**^		**0.964**^**c**^	**0.908**^**c**^	-0.093	**-0.833**^**c**^
	***COL3A1***	**0.585**^**a**^	**0.964**^**c**^		**0.904**^**c**^	-0.103	**-0.843**^**c**^
	***FBN1***	**0.527**^**a**^	**0.908**^**c**^	**0.904**^**c**^		0.080	**-0.764**^**c**^
	***FBN2***	0.122	-0.093	-0.103	0.080		0.090
	***FBN3***	-0.631^b^	**-0.833**^**c**^	**-0.843**^**c**^	**-0.764**^**c**^	0.090	
Steroidogenesis and growth factor signalling	***INSL3***	0.170	0.442	**0.508**^**a**^	0.463	**-0.503**^**a**^	**-0.509**^**a**^
	***CYP17A1***	-0.018	0.296	0.403	0.334	**-0.563**^**a**^	-0.336
	***CYP11A1***	0.094	0.291	0.401	0.305	**-0.516**^**a**^	-0.382
	***HSD3B1***	0.057	0.173	0.284	0.151	**-0.512**^**a**^	-0.296
	***LHCGR***	**-0.533**^**a**^	**-0.572**^**a**^	**-0.485**^**a**^	**-0.495**^**a**^	-0.172	**0.732**^**c**^
	***AR***	**0.650**^**b**^	**0.826**^**c**^	**0.842**^**c**^	**0.817**^**c**^	0.177	**-0.836**^**c**^
	***RARRES1***	0.454	**0.730**^**c**^	**0.638**^**b**^	**0.598**^**a**^	-0.090	**-0.572**^**a**^
	***IGFBP3***	0.366	**0.535**^**a**^	**0.558**^**a**^	0.460	0.004	**-0.600**^**a**^

As expected, the genes for enzymes of the androgen synthesis pathway, *CYP11A1*, *CYP17A1* and *HSD3B1*, were very strongly positively correlated (Table 3). *AR* showed a strong negative correlation with *LHCGR*, but a strong positive correlation with *IGFBP3*.

**Table 3:** Pearson’s correlation coefficients between transcript abundance levels of thecal cells and gestational age. The darker the background colour the more significant the correlation is. Blue indicates a negative correlation and red indicates a positive correlation. (*P*-values: a < 0.05, b < 0.01, c < 0.001).

		Steroidogenesis and growth factor signalling							
		*INSL3*	*CYP17A1*	*CYP11A1*	*HSD3B1*	*LHCGR*	*AR*	*RARRES1*	*IGFBP3*
Gestational age		**0.566**^**a**^	0.352	0.482	0.428	**-0.538**^**a**^	**0.788**^**c**^	**0.620**^**b**^	**0.490**^**a**^
Thecal Cells	***INSL3***		**0.886**^**c**^	**0.955**^**c**^	**0.904**^**c**^	0.063	0.275	0.196	0.211
	***CYP17A1***	**0.886**^**c**^		**0.932**^**c**^	**0.871**^**c**^	0.271	0.221	-0.109	0.336
	***CYP11A1***	**0.955**^**c**^	**0.932**^**c**^		**0.979**^**c**^	0.217	0.210	-0.023	0.209
	***HSD3B1***	**0.904**^**c**^	**0.871**^**c**^	**0.979**^**c**^		0.253	0.120	-0.097	0.147
	***LHCGR***	0.063	0.271	0.217	0.253		**-0.697**^**b**^	-0.455	-0.395
	***AR***	0.275	0.221	0.210	0.120	**-0.697**^**b**^		0.355	**0.679**^**b**^
	***RARRES1***	0.196	-0.109	-0.023	-0.097	-0.455	0.355		0.064
	***IGFBP3***	0.211	0.336	0.209	0.147	-0.395	**0.679**^**b**^	0.064	

### Transcript abundance relationships during gestation

**Fig 4:**
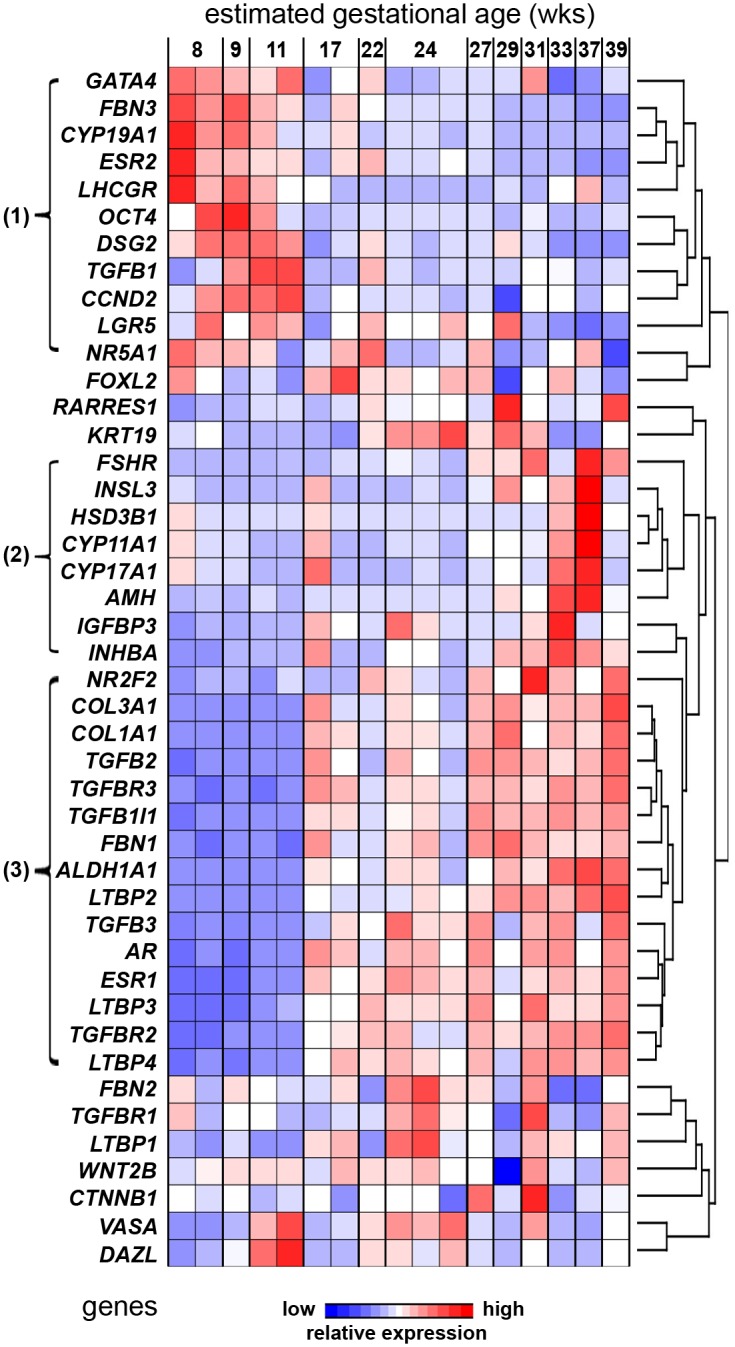
Heatmap of hierarchical clustering of transcript abundance in fetal ovaries throughout gestation. Hierarchical clustering analysis by transcript abundance only (rows) using q-PCR data of 44 genes (gene symbols on left) in n = 16–17 fetal ovaries (one per animal), from 8–39 weeks of gestation (left to right across the top). The raw data were first normalized by adjusting the mean expression across all samples for each gene to zero and the standard deviation to one. The genes were then clustered using the Euclidian algorithm for dissimilarity with average linkage in Partek Genomics Suite ™ (dendrogram on far right). Three transcript abundance clusters were identified as indicated by numbers and brackets on far left.

Hierarchical clustering of the expression levels throughout gestation of 24 genes associated with thecal and stromal cells and TGFβ signalling in this study with 20 genes associated with germ cells, GREL cells and granulosa cells in another study [19], revealed three distinct clusters (Fig 4). Cluster 1 contained genes which were highly expressed in the first trimester compared to the other two trimesters. This included the pluripotency marker *OCT4*, the stem cell marker *LGR5*, the desmosomal protein *DSG2*, genes connected with steroids, *CYP19A1*, *ESR2*, *NR5A1* and *LHCGR*, extracellular matrix associated genes *FBN3* and *TGFB1*, the cell cycle gene *CCND2*, and *GATA4*. Cluster 2 included genes which showed low expression during the first and second trimester and higher expression during the third trimester. These genes were associated with granulosa cells (*FSHR*, *INHBA*, *AMH* and *IGFBP3*) and thecal cells (*CYP17A1*, *CYP11A1*, *HSD3B1* and *INSL3*) of growing follicles. Genes of cluster 3 also showed very low expression in the first trimester and then the expression increased in trimester 2 and remained high until the end of gestation. The majority of genes in this cluster were associated with stroma (*NR2F2*, *COL3A1*, *COL1A1*, *FBN1*) and TGFβ-signalling (*TGFB2* and *3*, *LTBP2-4*, *TGFBR2* and *3*, *TGFB1I1*). Furthermore, the stem cell marker *ALDH1A1*, as well as the two steroidogenic receptors, *AR* and *ESR1*, had similar expression profiles. The remaining genes—germ cell markers *DAZL* and *VASA*, granulosa cell marker *FOXL2*, Wnt/β-catenin pathway genes *WNT2B* and *CTNNB1*, and *KRT19*, *RARRES1* and the stroma-related genes *FBN2*, *TGFBR1* and *LTBP1* showed no defined pattern of expression that was either similar to other genes nor specific for any particular phase/developmental stage. *DAZL* and *VASA* were, however, elevated briefly in week 11 samples.

**Fig 5:**
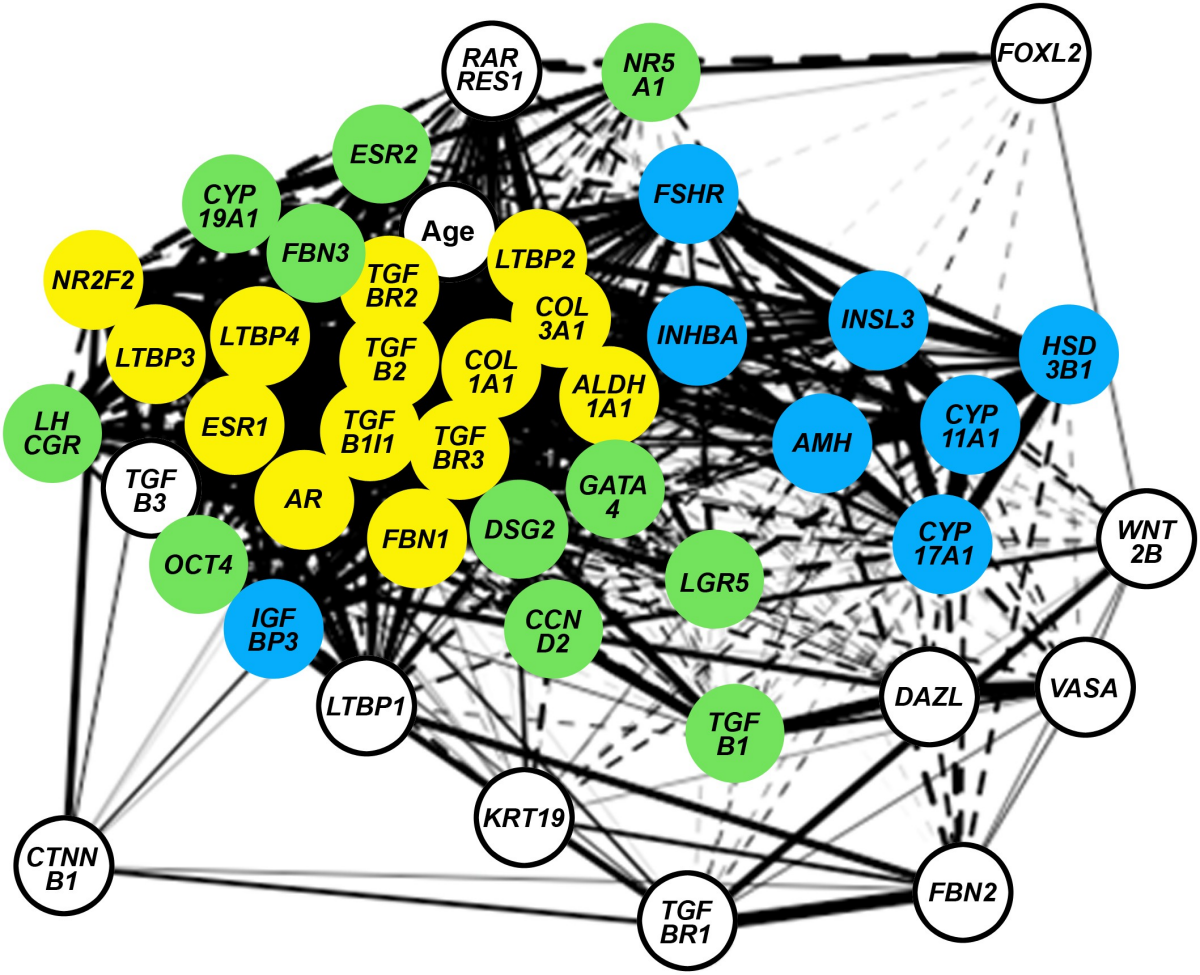
An adjacent matrix network graph using correlation coefficients from S2 Table. The closeness of the genes and the thickness of the interconnecting lines indicate stronger correlations between genes. Age is age of gestation. Green are cluster 1 genes, blue are cluster 2 genes and yellow are cluster 3 genes identified in Fig 4.

Correlation analyses were conducted on the expression levels of 24 genes associated thecal and stromal cells from this study with 20 genes associated with germ cells, GREL cells and granulosa cells in another study [27] and with day of gestation (S2 Table). After correlation values between genes were identified and regardless if they were positive or negative correlations, a network graph was plotted using the qgraph R package [28] using an adjacent matrix (Fig 5). Genes in each of clusters 2 and 3 were highly and closely correlated with each other whereas the genes in cluster 1 were less closely correlated with each other (Fig 5, S2 Table). Genes in clusters 1 and 2 were also closely, but negatively, correlated with each other (Fig 5, S2 Table).

## Discussion

The transcript abundance in whole ovaries of a number of genes in the developing bovine ovary were measured across gestation. Whilst we focused on 24 genes likely expressed in stroma we also compared these with 20 additional genes associated with germ cells, GREL cells and granulosa cells published previously [19]. We performed hierarchical clustering, and correlation and network analyses, looking for relationships between genes and gestational age. The genes associated into three clusters based upon their expression patterns, and new positive and negative relationships were identified.

Cluster 1 contained genes which were highly expressed in the first trimester compared to the last two trimesters. The genes in cluster 1 were of mixed function and were closely, but negatively, correlated with those in cluster 2 and were less closely correlated with each other than the genes in cluster 2 were. They included the pluripotency marker *OCT4*, the stem cell marker *LGR5*, the desmosomal protein *DSG2*, genes connected with steroid synthesis, action or regulation, such as *CYP19A1*, *ESR2*, and *LHCGR*, extracellular matrix associated genes *FBN3* and *TGFB1*, the cell cycle gene *CCND2*, and the transcription factors *NR5A1* and *GATA4* which are important for development and differentiation of cells in the ovary. During the first trimester when the cluster 1 genes are highly expressed the stroma is penetrating into the cortex from the medulla and is expanding at its fastest rate [3]. Both *NR5A1* and *FBN3* are expressed in the stromal cells [1,6]. It is possible that both of these genes and other genes in cluster 1 are involved in the stromal development.

Cluster 2 included genes which showed low expression during the first and second trimester, rising during the third trimester. These genes were associated with follicle formation, activation and maturation including those expressed in granulosa cells (*FSHR*, *INHBA*, *AMH* and *IGFBP3*), and in thecal cells (*CYP17A1*, *CYP11A1*, *HSD3B1* and *INSL3*). Thus, the cluster 2 genes clearly represent the periods when follicle activation and growth has commenced at least to the stage of thecal cell differentiation. Follicle growth clearly does not go beyond the thecal formation stage as there were no increases in *CYP19A1* in the third trimester.

Genes of cluster 3 showed very low expression in the first trimester which then increased in trimester 2 and remained high until the end of gestation. Members of this cluster have been shown to be localised to stroma and included transcription factors *AR* [29] and *NR2F2* [1], and the extracellular matrix genes *COL3A1*, *COL1A1* and *FBN1* [1]. They also included TGFβ-signalling genes (*TGFB2* and *3*, *LTBP2-4*, *TGFBR2* and *3*, *TGFB1I1*). Since TGFβ-signalling is important in stromal cell proliferation and collagen synthesis [30–34], these genes are likely involved in the steady expansion of stroma and the increase in collagen synthesis that occurs in the medulla and in the cortex of the ovary [3] at occurs during the latter part of gestation. Furthermore, the enzyme involved in retinoic acid synthesis, *ALDH1A1*, as well as the oestrogen receptor alpha, *ESR1*, showed similar expression profiles. *ALDH1A1* was a little different to the other cluster 3 genes in that if continued to rise in the third trimester.

These results provide valuable insights into fetal ovary development. The expression of AR and a number of TGFβ-signalling molecules in the current study were highly correlated with each other and formed cluster 3. Their expression first increased at day 119 a time when ovigerous cords are breaking down and follicles are beginning to form [27]. Potentially important is that one of the genes in cluster 3 is common to both AR and TGFβ-signalling pathways, *TGFB1I1* (TGFβ1 induced transcript 1 protein), also known as hic-5 and which is in fact an AR coactivator protein [35]. That these pathways intersect and have highly correlated levels of expression suggests that these signalling pathways are related and may in fact control the ovarian stroma in the latter part of gestation.

The remaining genes that were not part of the three gene clusters—germ cell markers *DAZL* and *VASA*, Wnt/β-catenin pathway genes *WNT2B* and *CTNNB1*, *KRT19*, *RARRES1* and the stroma-related genes *FBN2*, *TGFBR1* and *LTBP1*—showed no particular expression patterns similar to other genes nor specific for any particular phase/developmental stage.

The results could also provide insight into the fetal origins of PCOS. Three of the PCOS-associated genes [36] *FBN3*, *LHCGR* and *GATA4* in cluster 1 were highly positively correlated with each other and highly expressed in the first trimester. *FSHR* is also associated with PCOS, and was negatively correlated with these three genes and highly expressed with other follicle-associated genes in cluster 3 at the end of gestation. These relationships are illuminating. *GATA4* [37–41], *LHCGR* [42] and *FSHR* [43] are expressed and play important roles in adult ovaries and it is likely that their role in PCOS could be interpreted in the light of these adult functions. However, given that there is also a fetal origin of PCOS [44] it is possible that their contribution to the PCOS ovary phenotype is via their expression in the fetal ovary, as has been proposed for *FBN3* [6]. The other results that bear upon PCOS are those of expression of the AR and TGFβ-signalling. TGFβ-signalling is important for stimulating stromal growth and expression of collagens [30–34] and the PCOS ovarian phenotype is characterised by substantially increased cortical and medullary stroma and collagen [8,9]. TGFβ-signalling in the fetal ovary has been suggested previously to be involved in the PCOS ovary phenotype [6,44]. Androgen signalling and indeed AR expression have been demonstrated in numerous animal models to be important for developing a PCOS phenotype [16].

## Conclusions

The development of the ovary appears to undergo three major phases based upon transcript abundance. The early phase during which the stroma is actively replicating and expanding is characterised by expression of some genes related to PCOS, including *FBN3*, *LHCGR* and *GATA4*. In an intermediate stage the expression of stromal genes suggests that it has matured to express structural collagens and fibrillins associated with adult tissues, and under the control of TGFβ signalling and perhaps androgens. In the third phase the follicles have formed and some have commenced growth. The identification of the first two phases suggests that stroma is clearly dynamic during growth and development of the ovary and abnormalities may impact on the development of later reproductive pathologies.

## Supporting information

S1 Table Genes and forward (F) and reverse (R) primers used for qRT-PCR and the amplified product size. (PDF)

S2 Table Pearson’s Correlation Coefficients between all 44 genes related to specific cell types, structures and processes in fetal ovarian development as well as gestational age. The darker the background colour the more significant the correlation is. Blue indicates a negative correlation and red indicates a positive correlation. (*P*-values: a < 0.05, b < 0.01, c < 0.001, d < 0.0001).
(PDF)

S1 Fig TGF-β signalling pathway during trimesters of fetal ovarian development. Transcript abundance of markers specific for TGF-β signalling graphed by trimester (n = 16 and 17 animals respectively). Mean ± SEM are shown and statistical differences between trimesters are shown as *, **, ***, or ****, indicating *P* < 0.05, *P* < 0.01, *P* < 0.001 or *P* < 0.0001, respectively.
(TIF)

S2 Fig Extracellular matrix during trimesters of fetal ovarian development. Transcript abundance of markers of thecal steroidogenesis and growth factor signalling graphed by trimester (n = 16 and 17 animals respectively). Mean ± SEM are shown and statistical differences between trimesters are shown as **, ***, or ****, indicating *P* < 0.01, *P* < 0.001 or *P* < 0.0001, respectively.
(TIF)

S3 Fig Steroidogenesis and growth factor signalling during trimesters of fetal ovarian development. Transcript abundance of markers of thecal steroidogenesis and growth factor signalling graphed by trimester (n = 16 and 17 animals respectively). Mean ± SEM are shown and statistical differences between trimesters are shown as *, **, or ***, indicating *P* < 0.05, *P* < 0.01 or *P* < 0.001, respectively.
(TIF)

S4 Fig Scatter plots showing related mRNA expression levels of (A) *TGFB1I1* versus *AR*, (B) *FBN3* versus *AR* and (C) *FBN2* versus *TGFBR1* in whole bovine fetal ovaries (n = 16 and 17 animals respectively). Data are presented as normalised transcript abundance to *RPL32* and *PPIA*. Spearman’s correlation coefficient (R) test was used to analyse data.
(TIF)
