# Supplementary material for: Transcript abundance of stromal and thecal cell related genes during bovine ovarian development
Source: PLoS One. 2019 Mar 11;14(3):e0213575. doi: 10.1371/journal.pone.0213575 (PMC6411104; doi:10.1371/journal.pone.0213575)
Supplement: S1 Table — (PDF) [file pone.0213575.s001.pdf]

S1 Table. Genes and forward (F) and reverse (R) primers used for qRT-PCR and the amplified product size.

Gene name	Gene Symbol	GenBank Accession No.		Primers (5'-3')	Size (bp)
Androgen receptor	*AR*	NM_001244127.1	F:	TGCCCCTGACCTGGTTTTC	67
			R:	TCGGACACACTGGCTGTACATC	
Collagen, type I, alpha 1	*COL1A1*	NM_001034039.2	F:	TACTGGATTGACCCCAACCAAG	73
			R:	TCTCACCGGTTTCCATGTTG	
Collagen, type 3, alpha 1	*COL3A1*	NM_001076831.1	F:	TTCCCGTAAAAACCCTGCAC	146
			R:	ACGTTTCCCCAGTTTCCATG	
Cytochrome P450 family 11 subfamily A, member 1	*CYP11A1*	NM_176644.2	F:	CACTTTCGCCACATCGAGAA	85
			R:	TGAATGATATAAACTGACTCCAAATTG C	
Cytochrome P450, family 17, subfamily A, polypeptide 1	*CYP17A1*	NM_174304.2	F:	ACCATCAGAGAAGTGCTCCGAA	115
			R:	CCACAACGTCTGTGCCTTTGT	
Fibrillin 1	*FBN1*	NM_174053	F:	GGGATGGATTTTGTTCGAGGC	126
			R:	CATCACTGCAGCTACCTCCATT	
Fibrillin 2	*FBN2*	NM_001278588.1	F:	GGACTCCTACCAAGCAAGCATG	62
			R:	ACAAAGAACCCCATTCTGGATG	
Fibrillin 3	*FBN3*	XM_010806885.1	F:	GCCACAGCCTGCCTAGATGT	80
			R:	CTGCCCTCAGTGTTTTTGCA	
Glyceraldehyde 3-phosphate dehydrogenase	*GAPDH*	NM_001034034.2	F:	ACCACTTTGGCATCGTGGAG	76
			R:	GGGCCATCCACAGTCTTCTG	
Insulin growth factor binding protein 3	*IGFBP3*	NM_174556.1	F:	CGCCTGCGCCCTTACC	57
			R:	TTCTTCCGACTCACTGCCATT	
Insulin-like 3 (Leydig cell)	*INSL3*	NM_174365.2	F:	GGCGACCGTGAGCTCCT	62
			R:	ACTGGCCATCAGCCCATG	

Luteinizing hormone/ choriogonadotropin receptor	*LHCGR*	NM_174381.1	F:<br>R:	GCCACTGCTGTGCTTTTAGAAA<br>CCAGCCACTCAGTTCACTCTCA	158
Latent transforming growth factor beta binding protein 1	*LTBP1*	NM_001103091.1	F:<br>R:	GATTTGGGCCAGATCCTACCT<br>CGGTAACACGGCCCTTTCT	79
Latent transforming growth factor beta binding protein 2	*LTBP2*	NM_174385.2	F:<br>R:	CAGTCGAGTTCAGCGAGATCTG<br>TCTGTGTACGTGGTCTGTCCAAAC	88
Latent transforming growth factor beta binding protein 3	*LTBP3*	NM_001192738.1	F:<br>R:	CACTGCTGCCTTCAAGGAGATC<br>TCACTTTCGCCCTGAATGGT	90
Latent transforming growth factor beta binding protein 4	*LTBP4*	XM_010814978.1	F:<br>R:	CACCATGGCCGATGTACTGAT<br>CTTCGGTGTTAGTGCAATGGC	82
Nuclear receptor subfamily 2, group F, member 2	*NR2F2*	NM_174402.3	F:<br>R:	ACGGATCTTCCAAGAGCAAG<br>TTCCACATGGGCTACATCAG	130
Retinoic acid receptor responder (tazarotene induced) 1	*RARRES1*	NM_001075430.2	F:<br>R:	AAGCCCCTTGAATGCAGTCA<br>TGGGTCTCAGAGATGGAGCAA	65
Ribosomal protein L19	*RPL19*	NM_001040516.1	F:<br>R:	GATCCGGAAGCTGATCAAAG<br>TACCCATATGCCTGCCTTTC	113
Transforming growth factor beta 1	*TGFB1*	NM_001166068.1	F:<br>R:	CTGCTGAGGCTCAAGTTAAAAGTG<br>CAGCCGGTTGCTGAGGTAG	90
Transforming growth factor beta 1 induced transcript 1	*TGFB1I1*	NM_001035313.1	F:<br>R:	TCCCCTGTTCTCCCAAAGC<br>GCCCTGAGGCTGGAAGATG	109
Transforming growth factor beta 2	*TGFB2*	NM_001113252.1	F:<br>R:	AAGCCAGAGTGCCTGAACAAC<br>TTTCACGACTTTGCTGTCAATGT	101
Transforming growth factor beta 3	*TGFB3*	NM_001101183.1	F:<br>R:	TGCGTACCCCAGGACTTG<br>TGTTAGACAGCTGCTCCACTTTG	79

Transforming growth factor, beta receptor I	*TGFBR1*	NM_174621.2	F:<br>R:	CAGAGTGGGAACAAAAAGGTACATG<br>CATTGCATAGATGTCAGCACGTT	100
Transforming growth factor, beta receptor II (70/80kDa)	*TGFBR2*	NM_001159566.1	F:<br>R:	AACGTAGTAACTCCCTGCACTG<br>AAACAAACGTGGGTGTGACG	141
Transforming growth factor, beta receptor III	*TGFBR3*	XM_005204336.2	F:<br>R:	TGCACTTTCCTATCCCACAAGCCG<br>CCAGATCATTGAGGCATCCAGCG	197

F is forward and R is the reverse primer sequence.
