# Supplementary figures and images for: Transcript abundance of stromal and thecal cell related genes during bovine ovarian development
Source: PLoS One. 2019 Mar 11;14(3):e0213575. doi: 10.1371/journal.pone.0213575 (PMC6411104; doi:10.1371/journal.pone.0213575)

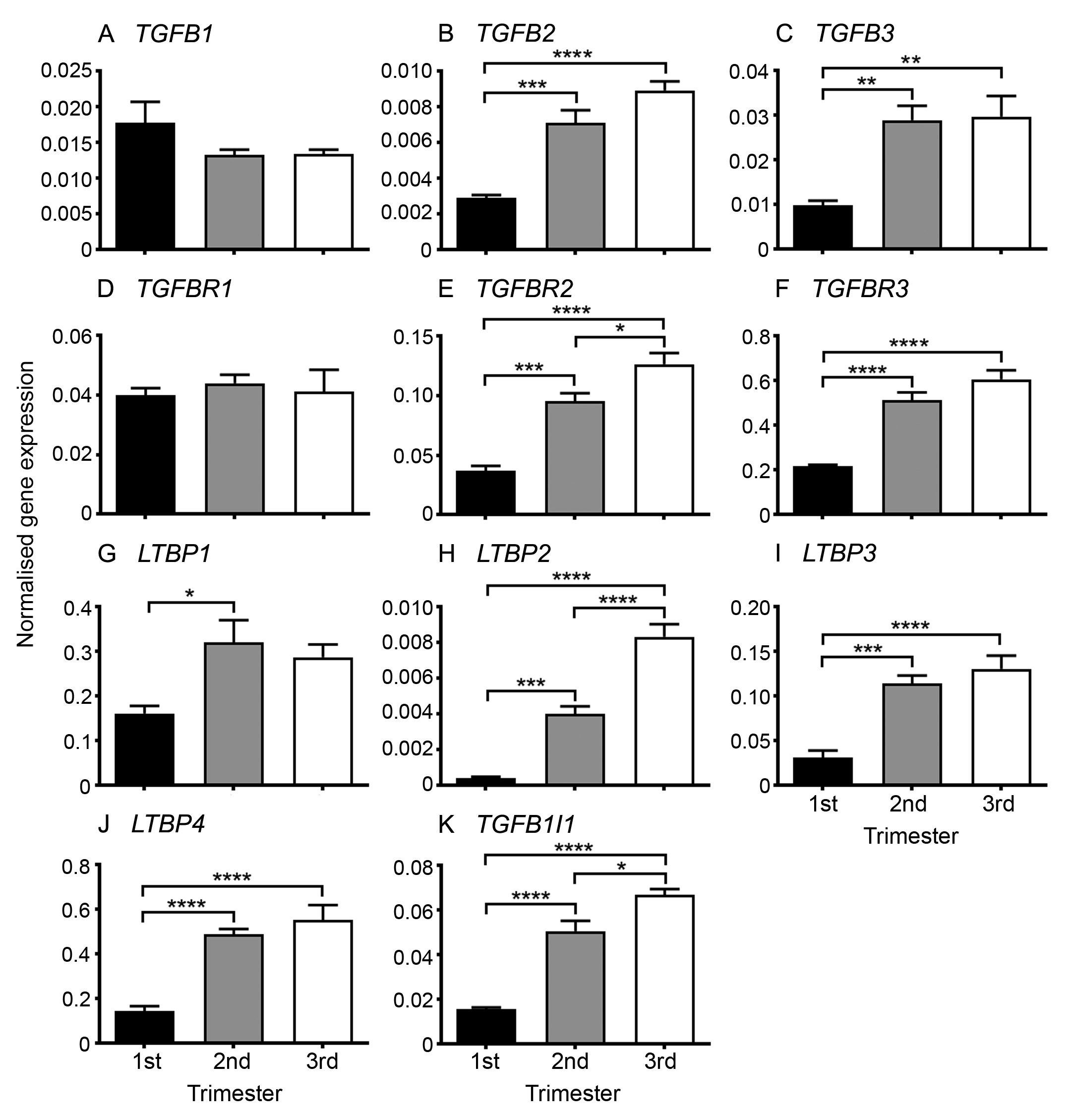

Supplement: S1 Fig — Transcript abundance of markers specific for TGF-β signalling graphed by trimester (n = 16 and 17 animals respectively). Mean ± SEM are shown and statistical differences between trimesters are shown as *, **, ***, or ****, indicating P < 0.05, P < 0.01, P < 0.001 or P < 0.0001, respectively. (TIF) [file pone.0213575.s003.tif]

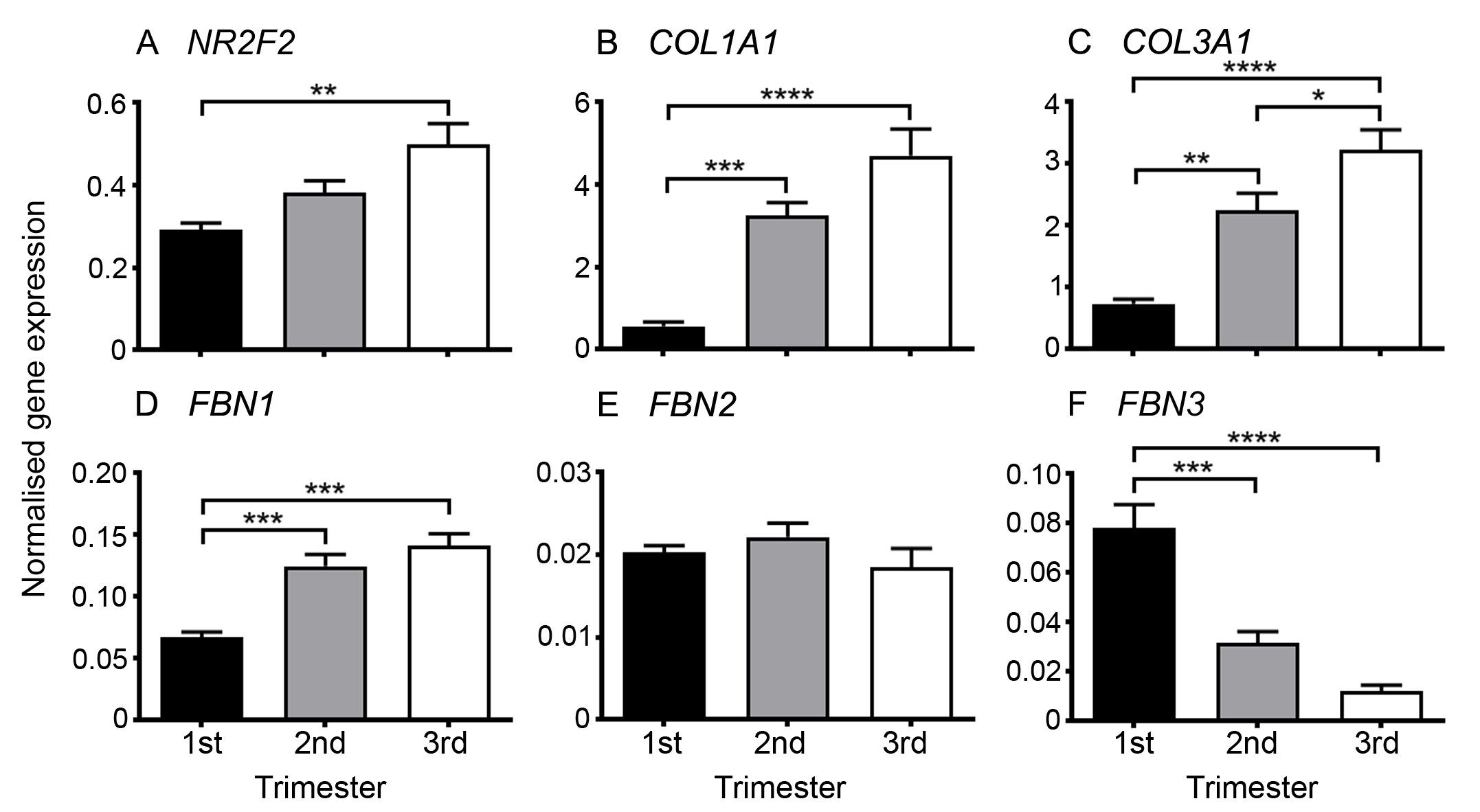

Supplement: S2 Fig — Transcript abundance of markers of thecal steroidogenesis and growth factor signalling graphed by trimester (n = 16 and 17 animals respectively). Mean ± SEM are shown and statistical differences between trimesters are shown as **, ***, or ****, indicating P < 0.01, P < 0.001 or P < 0.0001, respectively. (TIF) [file pone.0213575.s004.tif]

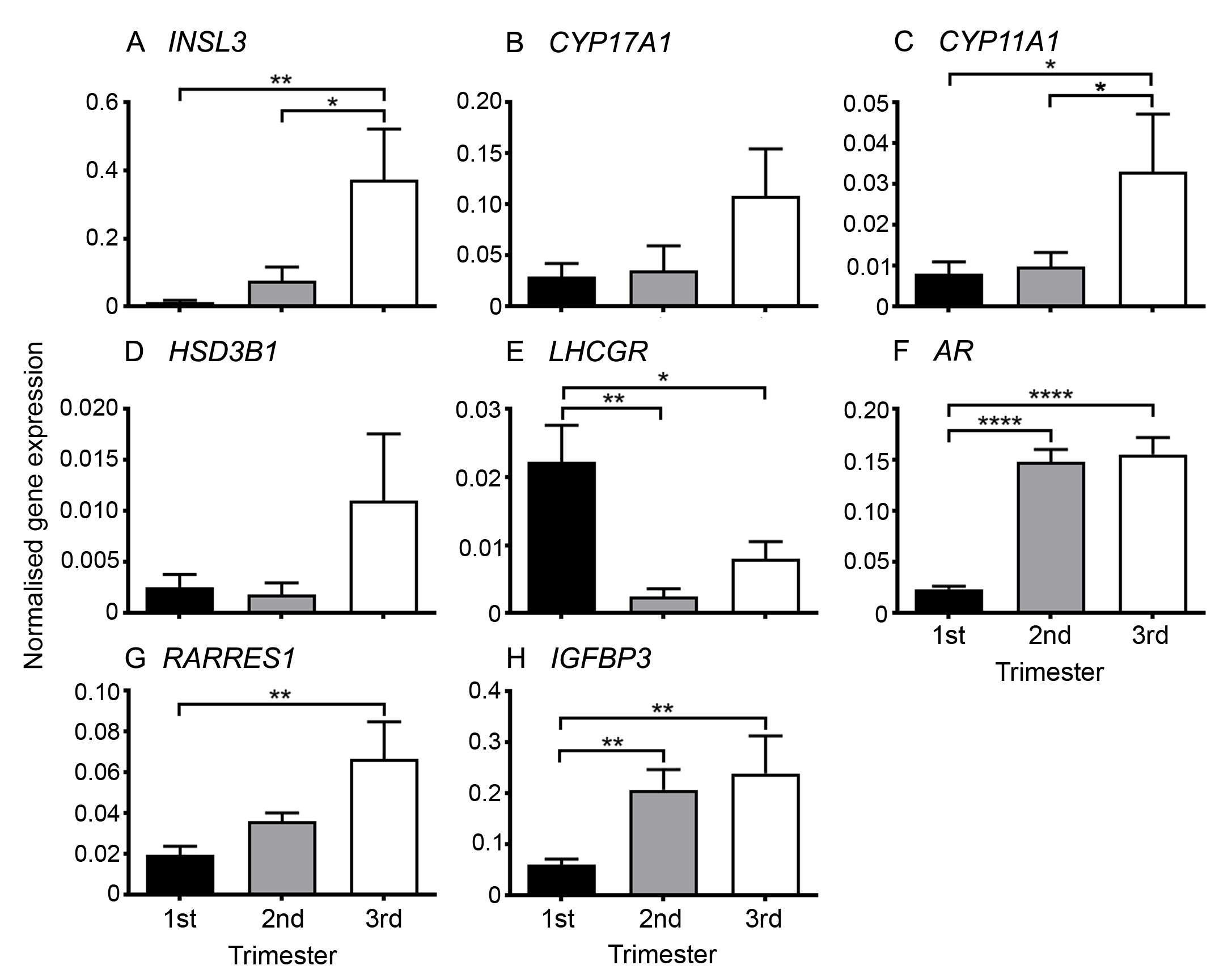

Supplement: S3 Fig — Transcript abundance of markers of thecal steroidogenesis and growth factor signalling graphed by trimester (n = 16 and 17 animals respectively). Mean ± SEM are shown and statistical differences between trimesters are shown as *, **, or ***, indicating P < 0.05, P < 0.01 or P < 0.001, respectively. (TIF) [file pone.0213575.s005.tif]

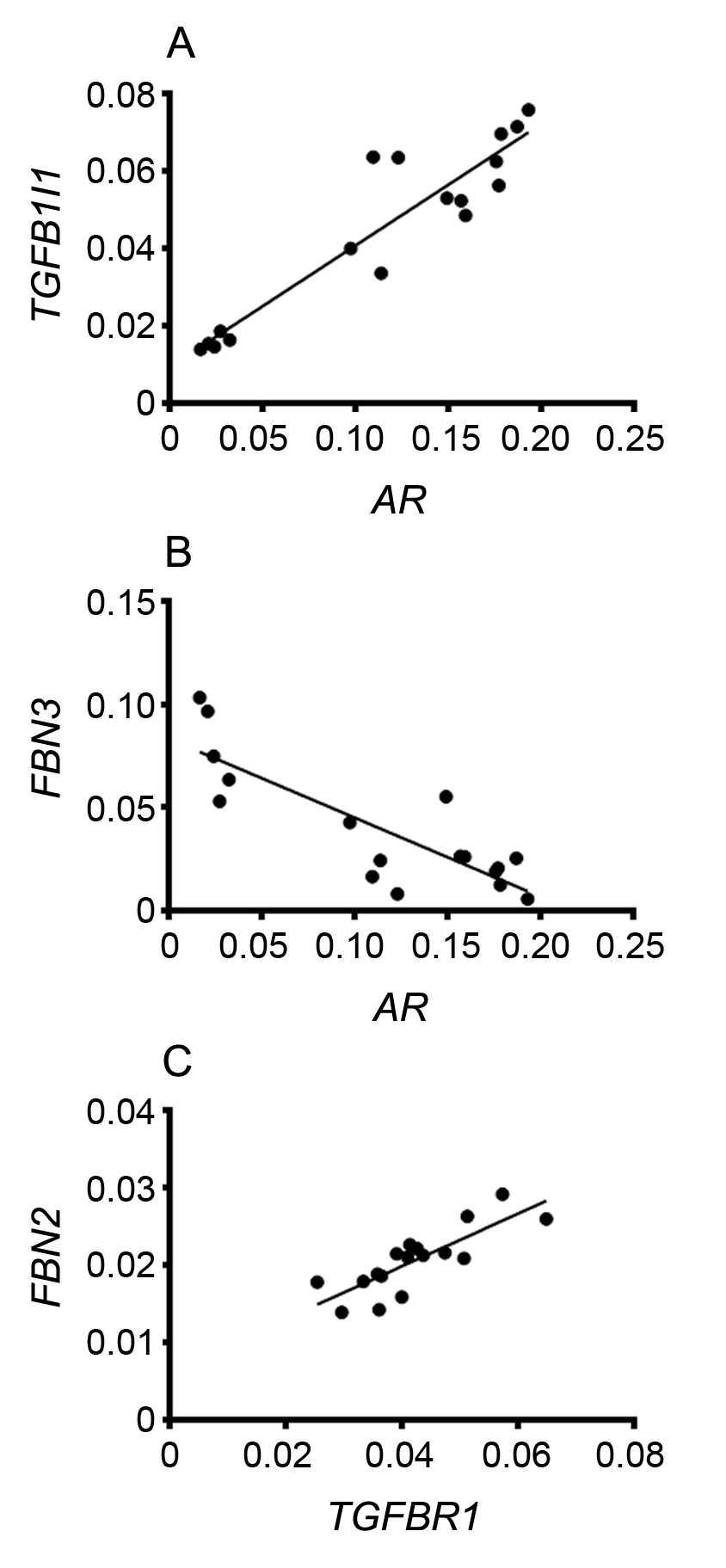

Supplement: S4 Fig — Data are presented as normalised transcript abundance to RPL32 and PPIA. Spearman’s correlation coefficient (R) test was used to analyse data. (TIF) [file pone.0213575.s006.tif]
