# Supplementary material for: Transcript abundance of stromal and thecal cell related genes during bovine ovarian development
Source: PLoS One. 2019 Mar 11;14(3):e0213575. doi: 10.1371/journal.pone.0213575 (PMC6411104; doi:10.1371/journal.pone.0213575)
Supplement: S2 Table — The darker the background colour the more significant the correlation is. Blue indicates a negative correlation and red indicates a positive correlation. (P-values: a < 0.05, b < 0.01, c < 0.001, d < 0.0001). (PDF) [file pone.0213575.s002.pdf]

S2 Table. Pearson's Correlation Coefficients between all 44 genes related to specific cell types, structures and processes in fetal ovarian development as well as gestational age. The darker the background colour the more significant the correlation is. Blue indicates a negative correlation and red indicates a positive correlation . (*P*-values: a < 0.05, b < 0.01, c < 0.001, d < 0.0001).

		Germ and stem cell markers					GREL cell markers		Granulosa cell markers												Stromal and TGF-β signalling markers											Extracellular Matrix						Thecal cell markers							
		OCT4	*DAZL*	*VASA*	*LGR5*	*ALDH1A1*	*KRT19*	*DSG2*	*FOXL2*	*AMH*	*FSHR*	*CYP19A1*	*ESR1*	*ESR2*	*INHBA*	*NR5A1*	*GATA4*	*WNT2B*	*CTNNB1*	*CCND2*	*TGFB1*	*TGFB2*	*TGFB3*	*TGFBR1*	*TGFBR2*	*TGFBR3*	*LTBP1*	*LTBP2*	*LTBP3*	*LTBP4*	*TGFB1I1*	*NF2R2*	*COL1A1*	*COL3A1*	*FBN1*	*FBN2*	*FBN3*	*INSL3*	*CYP17A1*	*CYP11A1*	*HSD3B1*	*LHCGR*	*AR*	*RARRES1*	*IGFBP3*
Gestational age		**-0.62**	-0.242	-0.022	**-0.479**	**0.897**	0.276	**-0.775**	-0.180	**0.618**	**0.733**	**0.794**	**0.822**	**-0.744**	**0.686**	-0.441	**-0.622**	-0.156	0.175	**-0.587**	-0.33	**0.818**[d]	**0.758**[c]	0.121	**0.838**[d]	**0.930**[d]	0.454	**0.957**[d]	**0.857**[d]	**0.855**[d]	**0.895**[d]	**0.754**[c]	**0.816**[d]	**0.843**[d]	**0.727**[c]	-0.114	**-0.890**[d]	**0.566**[a]	0.352	0.482	0.428	**-0.538**[a]	**0.788**[c]	**0.620**[b]	**0.490**[a]
Germ and stem cell markers	*OCT4*		0.055	-0.162	0.375	**-0.560**[a]	-0.197	**0.779**[c]	-0.160	-0.319	-0.314	**0.651**[b]	**-0.695**[b]	0.378	**-0.486**[a]	0.315	0.474	0.169	-0.039	**0.633**[b]	0.410	**-0.617**[b]	**-0.561**[a]	-0.038	**-0.651**[b]	**-0.694**[b]	-0.407	**-0.574**[a]	**-0.664**[b]	**-0.649**[b]	**-0.684**[b]	-0.372	**-0.657**[b]	**-0.605**[a]	**-0.618**[b]	0.061	**0.708**	-0.346	-0.291	-0.289	-0.219	**0.532**[a]	**-0.676**[b]	-0.352	-0.453
	DAZL	0.055		**0.858**[c]	0.284	-0.388	0.003	0.429	-0.368	-0.332	-0.215	-0.155	-0.140	0.008	-0.375	-0.381	0.346	0.319	-0.138	**0.597**[a]	**0.819**[c]	-0.405	-0.118	0.087	-0.256	-0.42	-0.249	-0.321	-0.093	-0.218	-0.402	-0.046	-0.365	-0.381	**-0.493**[a]	0.097	0.076	-0.397	-0.471	-0.412	-0.324	-0.183	-0.335	-0.027	-0.184
	VASA	-0.162	**0.858**[c]		0.191	-0.267	0.368	0.131	-0.196	-0.367	-0.093	-0.36	0.171	-0.137	-0.292	**-0.487**[a]	0.178	0.415	0.011	0.301	**0.532**[a]	-0.162	0.207	0.405	-0.055	-0.189	0.155	-0.111	0.203	0.074	-0.169	0.176	-0.185	-0.231	-0.249	0.418	-0.160	-0.419	**-0.561**[a]	-0.467	-0.395	**-0.483**[a]	-0.008	0.048	0.024
	LGR5	0.375	0.284	0.191		**-0.603**[a]	0.394	**0.714**[b]	-0.297	**-0.506**[a]	**-0.499**[a]	0.188	-0.476	0.351	**-0.573**[a]	0.053	0.432	-0.322	-0.204	0.098	0.272	-0.448	-0.419	-0.309	**-0.570**[a]	**-0.577**[a]	**-0.487**[b]	-0.465	-0.433	**-0.572**[a]	**-0.506**[a]	-0.372	-0.318	-0.471	-0.319	0.031	0.361	-0.437	**-0.586**[a]	**-0.567**[a]	**-0.573**[a]	-0.034	**-0.551**[a]	0.198	**-0.493**[a]
	ALDH1A1	**-0.560**[a]	-0.388	-0.267	**-0.603**[a]		-0.042	**-0.789**[c]	-0.137	**0.737**[c]	**0.702**[b]	**-0.645**[b]	**0.729**[c]	**-0.712**[b]	**0.795**[c]	-0.38	**-0.701**[b]	-0.212	0.011	**-0.512**[a]	-0.408	**0.808**[c]	**0.625**[b]	-0.050	**0.878**[c]	**0.878**[c]	0.471	**0.904**[c]	**0.660**[b]	**0.779**[c]	**0.866**[c]	**0.569**[a]	**0.838**[c]	**0.879**[c]	**0.703**[b]	-0.257	**-0.789**[c]	**0.695**[b]	**0.567**[a]	**0.643**[b]	**0.596**[a]	-0.345	**0.729**[c]	**0.517**[a]	**0.569**[a]
GREL cell markers	*KRT19*	-0.197	0.003	0.368	0.394	-0.042		-0.123	-0.208	-0.323	-0.018	-0.34	0.333	-0.146	-0.107	-0.343	-0.076	-0.204	0.171	**-0.525**[a]	-0.229	0.255	0.357	0.365	0.108	0.116	0.327	0.267	0.356	0.163	0.179	0.285	0.251	0.166	0.360	**0.504**[a]	-0.325	-0.235	-0.461	-0.379	-0.441	**-0.483**[a]	0.255	**0.497**[a]	0.045
	DSG2	**0.779**[c]	0.429	0.131	**0.714**[b]	**-0.789**[c]	-0.123		-0.277	**-0.517**[a]	**-0.507**[a]	**0.587**[a]	**-0.820**[c]	**0.549**[a]	**-0.673**[b]	0.270	**0.705**[b]	0.011	-0.100	**0.647**[b]	**0.661**[b]	**-0.765**[c]	**-0.697**[b]	-0.180	**-0.790**[c]	**-0.875**[c]	**-0.649**[b]	**-0.760**[c]	**-0.723**[c]	**-0.817**[c]	**-0.826**[c]	-0.467	**-0.723**[c]	**-0.761**[c]	**-0.703**[b]	0.021	**0.771**[c]	**-0.510**[a]	**-0.517**[a]	**-0.520**[a]	-0.457	0.436	**-0.859**[c]	-0.221	**-0.628**[b]
Granulosa cell markers	*FOXL2*	-0.16	-0.368	-0.196	-0.297	-0.137	-0.208	-0.277		-0.019	-0.248	0.240	0.110	0.306	-0.101	**0.598**[a]	-0.137	0.418	-0.062	-0.092	-0.43	-0.094	0.168	0.154	0.007	0.072	0.228	-0.284	-0.002	0.207	0.005	-0.206	-0.174	-0.189	-0.108	0.098	0.177	-0.162	0.073	-0.011	0.016	-0.016	0.213	**-0.623**[b]	0.21
	AMH	-0.319	-0.332	-0.367	**-0.506**[a]	**0.737**[c]	-0.323	**-0.517**[a]	-0.019		**0.624**[b]	-0.368	0.317	-0.481	**0.766**[c]	-0.011	**-0.563**[a]	-0.305	-0.13	-0.277	-0.229	0.370	0.224	-0.350	**0.556**[a]	0.464	0.096	0.573	0.272	0.432	0.504	0.262	0.399	0.452	0.300	**-0.576**[a]	-0.496	**0.857**[c]	**0.820**[c]	**0.872**[c]	**0.854**[c]	0.040	0.314	0.108	0.465
	FSHR	-0.314	-0.215	-0.093	**-0.499**[a]	**0.702**[b]	-0.018	**-0.507**[a]	-0.248	**0.624**[b]		-0.472	0.408	**-0.548**[a]	**0.520**[a]	-0.211	-0.259	-0.057	0.447	-0.314	-0.203	**0.566**[a]	0.369	0.118	**0.672**[b]	**0.494**[a]	0.231	**0.763**[c]	**0.573**[a]	**0.527**[a]	**0.598**[a]	**0.655**[b]	0.438	**0.525**[a]	0.452	-0.109	**-0.567**[a]	**0.737**[c]	**0.483**[a]	**0.692**[b]	**0.686**[b]	-0.156	0.425	0.308	0.107
	CYP19A1	**0.651**[b]	-0.155	-0.360	0.188	**-0.645**[b]	-0.34	**0.587**[a]	0.240	-0.368	-0.472		**-0.832**[c]	0.853	**-0.549**[a]	**0.581**[a]	**0.632**[b]	0.111	-0.085	0.439	0.017	**-0.715**[b]	**-0.675**[b]	0.003	**-0.784**[c]	**-0.717**[b]	-0.380	**-0.740**[c]	**-0.833**[c]	**-0.769**[c]	**-0.759**[c]	**-0.618**[b]	**-0.730**[c]	**-0.704**[b]	**-0.643**[b]	0.081	**0.941**[c]	-0.373	-0.138	-0.210	-0.151	**0.853**[c]	**-0.733**[c]	**-0.588**[a]	**-0.488**[a]
	ESR1	**-0.695**[b]	-0.140	0.171	-0.476	**0.729**[c]	0.333	**-0.820**[c]	0.110	0.317	0.408	**-0.832**[c]		-0.733	**0.550**[a]	-0.410	**-0.754**[c]	0.089	0.121	**-0.531**[a]	-0.321	**0.814**[c]	**0.910**[c]	0.254	**0.885**[c]	**0.873**[c]	**0.669**[b]	**0.736**[c]	**0.887**[c]	**0.933**[c]	**0.849**[c]	**0.636**[b]	**0.776**[c]	**0.803**[c]	**0.720**[b]	0.110	**-0.884**[c]	0.261	0.153	0.205	0.149	**-0.778**[c]	**0.928**[c]	0.408	**0.677**[b]
	ESR2	0.378	0.008	-0.137	0.351	**-0.712**[b]	-0.146	**0.549**[a]	0.306	-0.481	**-0.548**[a]	**0.853**[c]	**-0.733**[c]		**-0.721**[b]	**0.626**[b]	**0.748**[c]	0.152	-0.122	0.297	-0.005	**-0.778**[c]	**-0.575**[a]	0.026	**-0.730**[c]	**-0.737**[c]	-0.456	**-0.764**[c]	**-0.672**[b]	**-0.682**[c]	**-0.754**[c]	**-0.540**[a]	**-0.731**[c]	**-0.774**[c]	**-0.669**[b]	0.055	**0.878**[c]	**-0.510**[a]	-0.338	-0.366	-0.29	**0.619**[b]	**-0.718**[b]	-0.472	**-0.570**[a]
	INHBA	**-0.486**[a]	-0.375	-0.292	**-0.573**[a]	**0.795**[c]	-0.107	**-0.673**[b]	-0.101	**0.766**[c]	**0.520**[a]	**-0.549**[a]	**0.550**[b]	**-0.721**[b]		-0.339	**-0.699**[b]	-0.36	0.120	-0.424	-0.281	**0.719**[b]	0.416	-0.105	**0.680**[b]	**0.747**[c]	0.404	**0.722**[b]	**0.490**[a]	**0.587**[a]	**0.744**[c]	0.461	**0.707**[b]	**0.758**[c]	**0.685**[b]	-0.287	**-0.714**[b]	**0.696**[b]	**0.723**[b]	**0.625**[b]	**0.514**[a]	-0.199	**0.645**[b]	0.298	**0.752**[c]
	NR5A1	0.315	-0.381	**-0.487**[a]	0.053	-0.380	-0.343	0.27	0.598	-0.011	-0.211	**0.581**[a]	-0.410	**0.626**[b]	-0.339		0.240	0.118	0.036	0.068	-0.135	-0.468	-0.362	-0.167	-0.313	-0.408	-0.367	**-0.527**[a]	-0.372	-0.305	-0.385	-0.358	**-0.529**[a]	**-0.496**[a]	-0.359	-0.211	**0.584**[a]	-0.034	0.119	0.106	0.146	0.459	-0.377	**-0.609**[b]	-0.316
	GATA4	0.474	0.346	0.178	0.432	**-0.701**[c]	-0.076	**0.705**[b]	-0.137	**-0.563**[a]	-0.259	**0.632**[b]	**-0.754**[c]	**0.748**[c]	**-0.699**[b]	0.240		0.238	0.223	**0.512**[a]	0.351	**-0.674**[b]	**-0.539**[a]	0.164	**-0.637**[b]	**-0.754**[c]	**-0.560**[a]	**-0.595**[a]	-0.477	**-0.638**[b]	**-0.689**[b]	-0.156	**-0.683**[b]	**-0.723**[b]	**-0.657**[b]	0.134	**0.740**[c]	**-0.541**[a]	**-0.531**[a]	**-0.493**[a]	-0.413	0.441	**-0.717**[b]	-0.222	**-0.714**[b]
	WNT2B	0.169	0.319	0.415	-0.322	-0.212	-0.204	0.011	0.418	-0.305	-0.057	0.111	0.089	0.152	-0.36	0.118	0.238		0.185	**0.555**[a]	0.231	-0.265	0.241	**0.641**[b]	-0.023	-0.138	0.237	-0.254	0.127	0.198	-0.179	0.161	-0.401	-0.323	-0.436	0.403	0.185	-0.477	-0.374	-0.315	-0.184	-0.135	0.066	**-0.515**[a]	0.004
	CTNNB1	-0.039	-0.138	0.011	-0.204	0.011	0.171	-0.100	-0.062	-0.13	0.447	-0.085	0.121	-0.122	0.120	0.036	0.223	0.185		-0.052	-0.029	0.325	0.238	**0.508**[a]	0.240	0.142	0.173	0.213	0.431	0.212	0.279	**0.625**[b]	0.069	0.158	0.331	0.402	-0.117	0.013	-0.053	-0.018	-0.085	-0.097	0.278	-0.036	-0.074
	CCND2	**0.633**[b]	**0.597**[a]	0.301	0.098	**-0.512**[a]	**-0.525**[a]	**0.647**[b]	-0.092	-0.277	-0.314	0.439	**-0.531**[a]	0.297	-0.424	0.068	**0.512**[a]	**0.555**[a]	-0.052		**0.742**[c]	**-0.653**[b]	-0.366	0.089	**-0.519**[a]	**-0.621**[b]	-0.332	**-0.623**[b]	**-0.499**[a]	-0.449	**-0.632**[b]	-0.251	**-0.686**[b]	**-0.618**[b]	**-0.785**[c]	0.052	**0.570**[a]	-0.453	-0.31	-0.325	-0.207	0.355	**-0.560**[a]	**-0.532**[a]	-0.246
Stromal and TGF-β signalling markers	*TGFB1*	0.410	**0.819**[c]	**0.532**[a]	0.272	-0.408	-0.229	**0.661**[b]	-0.43	-0.229	-0.203	0.017	-0.321	-0.005	-0.281	-0.135	0.351	0.231	-0.029	**0.742**[c]		-0.441	-0.282	-0.033	-0.298	**-0.499**[a]	-0.418	-0.395	-0.228	-0.334	-0.452	-0.049	-0.436	-0.398	**-0.503**[a]	-0.039	0.251	-0.319	-0.357	-0.338	-0.269	0.06	-0.455	-0.094	-0.224
	TGFB2	**-0.617**[b]	-0.405	-0.162	-0.448	**0.808**[c]	0.255	**-0.765**[c]	-0.094	0.370	**0.566**[a]	**-0.715**[b]	**0.814**[c]	**-0.778**[c]	**0.719**[b]	-0.468	**-0.674**[b]	-0.265	0.325	**-0.653**[b]	-0.441		**0.720**[b]	0.092	**0.861**[c]	**0.945**[c]	**0.575**[a]	**0.863**[c]	**0.796**[c]	**0.796**[c]	**0.954**[c]	**0.652**[b]	**0.944**[c]	**0.959**[c]	**0.945**[c]	0.057	**-0.837**[c]	0.462	0.323	0.333	0.21	**-0.570**[a]	**0.898**[c]	**0.579**[a]	**0.541**[a]
	TGFB3	**-0.561**[a]	-0.118	0.207	-0.419	**0.625**[b]	0.357	**-0.697**[b]	0.168	0.224	0.369	**-0.675**[b]	**0.910**[c]	**-0.575**[a]	0.416	-0.362	**-0.539**[a]	0.241	0.238	-0.366	-0.282	**0.720**[b]		0.418	**0.832**[c]	**0.764**[c]	**0.665**[b]	**0.630**[b]	**0.850**[c]	**0.920**[c]	**0.776**[c]	**0.740**[c]	**0.661**[b]	**0.676**[b]	**0.572**[a]	0.249	**-0.726**[c]	0.041	-0.063	0.026	-0.006	**-0.717**[b]	**0.868**[c]	0.336	**0.646**[b]
	TGFBR1	-0.038	0.087	0.405	-0.309	-0.050	0.365	-0.180	0.154	-0.350	0.118	0.003	0.254	0.026	-0.105	-0.167	0.164	**0.641**[b]	**0.508**[a]	0.089	-0.033	0.092	0.418		0.110	0.076	**0.604**[a]	0.107	0.34	0.279	0.082	0.443	-0.077	-0.017	0.044	**0.815**[c]	-0.034	-0.413	-0.411	-0.356	-0.321	-0.159	0.298	-0.108	0.156
	TGFBR2	**-0.651**[b]	-0.256	-0.055	**-0.570**[a]	**0.878**[c]	0.108	**-0.790**[c]	0.007	**0.556**[a]	**0.672**[b]	**-0.784**[c]	**0.885**[c]	**-0.730**[c]	**0.680**[b]	-0.313	**-0.637**[b]	-0.023	0.24	**-0.519**[a]	-0.298	**0.861**[c]	**0.832**[c]	0.110		**0.900**[c]	0.457	**0.857**[c]	**0.902**[c]	**0.943**[c]	**0.928**[c]	**0.786**[c]	**0.822**[c]	**0.863**[c]	**0.735**[c]	-0.147	**-0.856**[c]	**0.489**[a]	0.338	0.428	0.375	**-0.607**[b]	**0.866**[c]	**0.489**[a]	**0.567**[a]
	TGFBR3	**-0.694**[b]	-0.42	-0.189	**-0.577**[a]	**0.878**[c]	0.116	**-0.875**[c]	0.072	0.464	**0.494**[a]	**-0.717**[b]	**0.873**[c]	**-0.737**[c]	**0.747**[c]	-0.408	**-0.754**[c]	-0.138	0.142	**-0.621**[b]	**-0.499**[a]	**0.945**[c]	**0.764**[c]	0.076	**0.900**[c]		**0.617**[b]	**0.849**[c]	**0.801**[c]	**0.883**[c]	**0.968**[c]	**0.590**[a]	**0.936**[c]	**0.946**[c]	**0.870**[c]	-0.043	**-0.844**[c]	0.459	0.39	0.379	0.284	**-0.579**[a]	**0.942**[c]	**0.490**[a]	**0.646**[b]
	LTBP1	-0.407	-0.249	0.155	**-0.487**[b]	0.471	0.327	**-0.649**[b]	0.228	0.096	0.231	-0.380	**0.669**[b]	-0.456	0.404	-0.367	**-0.560**[a]	0.237	0.173	-0.332	-0.418	**0.575**[a]	**0.665**[b]	**0.604**[a]	0.457	**0.617**[b]		0.469	**0.497**[a]	**0.617**[b]	**0.554**[a]	0.352	0.479	**0.488**[a]	**0.533**[a]	**0.632**[b]	**-0.485**[a]	0.063	0.034	0.047	0.018	-0.425	**0.720**[b]	0.082	**0.646**[b]
	LTBP2	**-0.574**[a]	-0.321	-0.111	-0.465	**0.904**[c]	0.267	**-0.760**[c]	-0.284	0.573	**0.763**[c]	**-0.740**[c]	**0.736**[c]	**-0.764**[c]	**0.722**[b]	**-0.527**[a]	**-0.595**[a]	-0.254	0.213	**-0.623**[b]	-0.395	**0.863**[c]	**0.630**[b]	0.107	**0.857**[c]	**0.849**[c]	0.469		**0.784**[c]	**0.758**[c]	**0.893**[c]	**0.691**[b]	**0.859**[c]	**0.884**[c]	**0.799**[c]	-0.071	**-0.868**[c]	**0.624**[a]	0.407	**0.508**[a]	0.433	-0.459	**0.762**[c]	**0.680**[b]	0.416
	LTBP3	**-0.664**[b]	-0.093	0.203	-0.433	**0.660**[b]	0.356	**-0.723**[c]	-0.002	0.272	**0.573**[a]	**-0.833**[c]	**0.887**[c]	**-0.672**[b]	**0.490**[a]	-0.372	-0.477	0.127	0.431	**-0.499**[a]	-0.228	**0.796**[c]	**0.850**[c]	0.34	**0.902**[c]	**0.801**[c]	**0.497**[a]	**0.784**[c]		**0.916**[c]	**0.864**[c]	**0.855**[c]	**0.718**[b]	**0.737**[c]	**0.712**[b]	0.113	**-0.842**[c]	0.250	0.069	0.16	0.102	**-0.769**[c]	**0.874**[c]	0.48	0.441
	LTBP4	**-0.649**[b]	-0.218	0.074	**-0.572**[a]	**0.779**[c]	0.163	**-0.817**[c]	0.207	0.432	**0.527**[a]	**-0.769**[c]	**0.933**[c]	**-0.682**[c]	**0.587**[a]	-0.305	**-0.638**[b]	0.198	0.212	-0.449	-0.334	**0.796**[c]	**0.920**[c]	0.279	**0.943**[c]	**0.883**[c]	**0.617**[b]	**0.758**[c]	**0.916**[c]		**0.891**[c]	**0.752**[c]	**0.745**[c]	**0.761**[c]	**0.655**[b]	0.038	**-0.821**[c]	0.294	0.187	0.257	0.223	**-0.719**[b]	**0.930**[c]	0.348	**0.648**[b]
	TGFB1I1	**-0.684**[b]	-0.402	-0.169	**-0.506**[a]	**0.866**[c]	0.179	**-0.826**[c]	0.005	0.504	**0.598**[a]	**-0.759**[c]	**0.849**[c]	**-0.754**[c]	**0.744**[c]	-0.385	**-0.689**[b]	-0.179	0.279	**-0.632**[b]	-0.452	**0.954**[c]	**0.776**[c]	0.082	**0.928**[c]	**0.968**[c]	**0.554**[a]	**0.893**[c]	**0.864**[c]	**0.891**[c]		**0.695**[b]	**0.926**[c]	**0.930**[c]	**0.887**[c]	-0.036	**-0.866**[c]	**0.498**[a]	0.365	0.395	0.293	**-0.597**[a]	**0.929**[c]	**0.537**[a]	**0.568**[a]
Extracellular Matrix	*NF2R2*	-0.372	-0.046	0.176	-0.372	**0.569**[a]	0.285	-0.467	-0.206	0.262	**0.655**[b]	**-0.618**[b]	**0.636**[b]	**-0.540**[a]	0.461	-0.358	-0.156	0.161	**0.625**[b]	-0.251	-0.049	**0.652**[b]	**0.740**[c]	0.443	**0.786**[c]	**0.590**[a]	0.352	**0.691**[b]	**0.855**[c]	**0.752**[c]	**0.695**[b]		**0.543**[a]	**0.585**[a]	**0.527**[a]	0.122	**-0.631**[b]	0.17	-0.018	0.094	0.057	**-0.533**[a]	**0.650**[b]	0.454	0.366
	COL1A1	**-0.657**[b]	-0.365	-0.185	-0.318	**0.838**[c]	0.251	**-0.723**[c]	-0.174	0.399	0.438	**-0.730**[c]	**0.776**[c]	**-0.731**[c]	**0.707**[b]	**-0.529**[a]	**-0.683**[b]	-0.401	0.069	**-0.686**[b]	-0.436	**0.944**[c]	**0.661**[b]	-0.077	**0.822**[c]	**0.936**[c]	**0.479**	**0.859**[c]	**0.718**[b]	**0.745**[c]	**0.926**[c]	**0.543**[a]		**0.964**[c]	**0.908**[c]	-0.093	**-0.833**[c]	0.442	0.296	0.291	0.173	**-0.572**[a]	**0.826**[c]	**0.730**[b]	**0.535**[a]
	COL3A1	**-0.605**[a]	-0.381	-0.231	-0.471	**0.879**[c]	0.166	**-0.761**[c]	-0.189	0.452	**0.525**[a]	**-0.704**[b]	**0.803**[c]	**-0.774**[c]	**0.758**[c]	**-0.496**[a]	**-0.723**[b]	-0.323	0.158	**-0.618**[b]	-0.398	**0.959**[c]	**0.676**[b]	-0.017	**0.863**[c]	**0.946**[c]	**0.488**[a]	**0.884**[c]	**0.737**[c]	**0.761**[c]	**0.930**[c]	**0.585**[a]	**0.964**[c]		**0.904**[c]	-0.103	**-0.843**[c]	**0.508**[a]	0.403	0.401	0.284	**-0.485**[a]	**0.842**[c]	**0.638**[b]	**0.558**[a]
	FBN1	**-0.618**[b]	**-0.493**[a]	-0.249	-0.319	**0.703**[b]	0.360	**-0.703**[b]	-0.108	0.300	0.452	**-0.643**[b]	**0.720**[b]	**-0.669**[b]	**0.685**[b]	-0.359	**-0.657**[b]	-0.436	0.331	**-0.785**[c]	**-0.503**[a]	**0.945**[c]	**0.572**[a]	0.044	**0.735**[c]	**0.870**[c]	**0.533**[a]	**0.799**[c]	**0.712**[b]	**0.655**[b]	**0.887**[c]	**0.527**[a]	**0.908**[c]	**0.904**[c]		0.080	**-0.764**[c]	0.463	0.334	0.305	0.151	**-0.495**[a]	**0.817**[c]	**0.598**[a]	0.46
	FBN2	0.061	0.097	0.418	0.031	-0.257	**0.504**[a]	0.021	0.098	**-0.576**[a]	-0.109	0.081	0.110	0.055	-0.287	-0.211	0.134	0.403	0.402	0.052	-0.039	0.057	0.249	**0.815**[c]	-0.147	-0.043	**0.632**[b]	-0.071	0.113	0.038	-0.036	0.122	-0.093	-0.103	0.080		0.090	**-0.503**[a]	**-0.563**[a]	**-0.516**[a]	**-0.512**[a]	-0.172	0.177	-0.090	0.004
	FBN3	**0.708**[b]	0.076	-0.160	0.361	**-0.789**[c]	-0.325	**0.771**[c]	0.177	-0.496	**-0.567**[a]	**0.941**[c]	**-0.884**[c]	**0.878**[c]	**-0.714**[b]	**0.584**[a]	**0.740**[c]	0.185	-0.117	**0.570**[a]	0.251	**-0.837**[c]	**-0.726**[c]	-0.034	**-0.856**[c]	**-0.844**[c]	**-0.485**[a]	**-0.868**[c]	**-0.842**[c]	**-0.821**[c]	**-0.866**[c]	-0.631[b]	**-0.833**[c]	**-0.843**[c]	**-0.764**[c]	0.090		**-0.509**[a]	-0.336	-0.382	-0.296	**0.732**[c]	**-0.836**[c]	**-0.572**[a]	**-0.600**[a]
Thecal cell markers	*INSL3*	-0.346	-0.397	-0.419	-0.437	**0.695**[b]	-0.235	**-0.510**[a]	-0.162	**0.857**[c]	**0.737**[c]	-0.373	0.261	**-0.510**[a]	**0.696**[b]	-0.034	**-0.541**[a]	-0.477	0.013	-0.453	-0.319	0.462	0.041	-0.413	**0.489**[a]	0.459	0.063	**0.624**[b]	0.25	0.294	**0.498**[a]	0.17	0.442	**0.508**[a]	0.463	**-0.503**[a]	**-0.509**[a]		**0.886**[c]	**0.955**[c]	**0.904**[c]	0.063	0.275	0.196	0.211
	CYP17A1	-0.291	-0.471	**-0.561**[a]	**-0.586**[a]	**0.567**[a]	-0.461	**-0.517**[a]	0.073	**0.820**[c]	**0.483**[a]	-0.138	0.153	-0.338	**0.723**[b]	0.119	**-0.531**[a]	-0.374	-0.053	-0.31	-0.357	0.323	-0.063	-0.411	0.338	0.39	0.034	0.407	0.069	0.187	0.365	-0.018	0.296	0.403	0.334	**-0.563**[a]	-0.336	**0.886**[c]		**0.932**[c]	**0.871**[c]	0.271	0.221	-0.109	0.336
	CYP11A1	-0.289	-0.412	-0.467	**-0.567**[a]	**0.643**[b]	-0.379	**-0.520**[a]	-0.011	**0.872**[c]	**0.692**[b]	-0.210	0.205	-0.366	**0.625**[b]	0.106	**-0.493**[a]	-0.315	-0.018	-0.325	-0.338	0.333	0.026	-0.356	0.428	0.379	0.047	**0.508**[a]	0.16	0.257	0.395	0.094	0.291	0.401	0.305	**-0.516**[a]	-0.382	**0.955**[c]	**0.932**[c]		**0.979**[c]	0.217	0.21	-0.023	0.209
	HSD3B1	-0.219	-0.324	-0.395	**-0.573**[a]	**0.596**[a]	-0.441	-0.457	0.016	**0.854**[c]	**0.686**[b]	-0.151	0.149	-0.29	**0.514**[a]	0.146	-0.413	-0.184	-0.085	-0.207	-0.269	0.210	-0.006	-0.321	0.375	0.284	0.018	0.433	0.102	0.223	0.293	0.057	0.173	0.284	0.151	**-0.512**[a]	-0.296	**0.904**[c]	**0.871**[c]	**0.979**[c]		0.253	0.12	-0.097	0.147
	LHCGR	**0.532**[a]	-0.183	**-0.483**[a]	-0.034	-0.345	**-0.483**[a]	0.436	-0.016	0.040	-0.156	**0.853**[c]	**-0.778**[c]	**0.619**[b]	-0.199	0.459	0.441	-0.135	-0.097	0.355	0.060	**-0.570**[a]	**-0.717**[b]	-0.159	**-0.607**[b]	**-0.579**[a]	-0.425	-0.459	**-0.769**[c]	**-0.719**[b]	**-0.597**[a]	**-0.533a**	**-0.572**[a]	**-0.485**[a]	**-0.495**[a]	-0.172	**0.732**[c]	0.063	0.271	0.217	0.253		**-0.697**[b]	-0.455	-0.395
	AR	**-0.676**[b]	-0.335	-0.008	**-0.551**[a]	**0.729**[c]	0.255	**-0.859**[c]	0.213	0.314	0.425	**-0.733**[c]	**0.928**[c]	**-0.718**[b]	**0.645**[b]	-0.377	**-0.717**[b]	0.066	0.278	**-0.560**[a]	-0.455	**0.898**[c]	**0.868**[c]	0.298	**0.866**[c]	**0.942**[c]	**0.720**[b]	**0.762**[c]	**0.874**[c]	**0.930**[c]	**0.929**[c]	**0.650**[b]	**0.826**[c]	**0.842**[c]	**0.817**[c]	0.177	**-0.836**[c]	0.275	0.221	0.21	0.12	**-0.697**[b]		0.355	**0.679**[b]
	RARRES1	-0.352	-0.027	0.048	0.198	**0.517**[a]	**0.497**[a]	-0.221	**-0.623**[b]	0.108	0.308	**-0.588**[a]	0.408	-0.472	0.298	**-0.609**[b]	-0.222	**-0.515**[a]	-0.036	**-0.532**[a]	-0.094	**0.579**[a]	0.336	-0.108	**0.489**[a]	**0.490**[a]	0.082	**0.680**[b]	0.48	0.348	**0.537**[a]	0.454	**0.730**[c]	**0.638**[b]	**0.598**[a]	-0.09	**-0.572**[a]	0.196	-0.109	-0.023	-0.097	-0.455	0.355		0.064
	IGFBP3	-0.453	-0.184	0.024	**-0.493**[a]	**0.569**[a]	0.045	**-0.628**[b]	0.210	0.465	0.107	**-0.488**[a]	**0.677**[b]	**-0.570**[a]	**0.752**[c]	-0.316	**-0.714**[b]	0.004	-0.074	-0.246	-0.224	**0.541**[a]	**0.646**[b]	0.156	**0.567**[a]	**0.646**[b]	**0.646**[b]	0.416	0.441	**0.648**[b]	**0.568**[a]	0.366	**0.535**[a]	**0.558**[a]	0.460	0.004	**-0.600**[a]	0.211	0.336	0.209	0.147	-0.395	**0.679**[b]	0.064	
